# A Comparative Analysis of Conductance Probes and High-Speed Camera Measurements for Interfacial Behavior in Annular Air–Water Flow

**DOI:** 10.3390/s23208617

**Published:** 2023-10-21

**Authors:** Yago Rivera, Maxime Bidon, José-Luis Muñoz-Cobo, Cesar Berna, Alberto Escrivá

**Affiliations:** 1 IIE Instituto de Ingeniería Energética, Universitat Politécnica de Valencia, 46022 Valencia, Spain; yaridu@upv.es (Y.R.); ceberes@iie.upv.es (C.B.); aescriva@iqn.upv.es (A.E.); 2 ENSEA—École Nationale Supérieur d’Électronique et de ses Applications, 95000 Cergy, France; maxime.bidon@ensea.fr

**Keywords:** conductance probes, annular flow, high-speed camera measurements, laser-induced fluorescence measurements

## Abstract

Different techniques are used to analyze annular flow, but the more interesting ones are those techniques that do not perturb the flow and provide enough resolution to clearly distinguish the interfacial phenomena that take place at the interface, especially the disturbance waves (DW) and the ripple waves (DW). The understanding of these events is important because it influences the heat and mass transfer taking place through the thin film formed near the walls in this flow regime. The laser-induced fluorescence (LIF) and the three-electrode conductance probe are two commonly used techniques to study experimentally annular flow phenomena. In this paper, a set of experiments at different temperatures of 20 °C, 30 °C and 40 °C and different liquid Reynolds numbers have been performed in the annular flow regime, the characteristic of the DW and RW as average height and frequency of these waves has been measured by both techniques LIF and conductance probes. In addition, we also measured the mean film thickness. It was found that the mean film thickness and the DW height are practically the same when measured by both techniques; however, the height of the RW is smaller when measured by the conductance probe and this difference diminishes when the temperature increases.

## 1. Introduction

Annular flow is one of the most important two-phase flow regimes because it is the dominant regime for heat transfer in refrigeration equipment, boiling water reactors (BWR), passive safety systems as the passive containment cooling condensers in the simplified BWR and some types of small modular reactors (SMR) [1,2,3,4]. Also, this transfer regime appears in the chemical industry and in classical thermal plants that use combined cycles [5]. The purpose of this paper is to perform a comparative analysis of two of the main measurement methods used to obtain the main characteristics of annular flow and the waves produced at the liquid and gas phases interface. These two methods are the conductance probe method and the high-speed camera measurement method; each one has its own advantages and disadvantages that will be discussed in this paper [6,7] and compared experimentally. As it is well known, annular flow is characterized by a small amount of liquid moving close to the walls and forming an annular film while the gas is moving through the core of this annulus [8]. Generally, depending on the relative velocity of the liquid film and the gas core, waves of different types are formed at the interface between the two phases. In addition, the shear stress at the interface can tear off small drops from the crest of these waves, which are then dragged by the core gas flow. In this regime, the liquid film contains small bubbles of gas, while the core gas flow drags small droplets previously separated from the liquid film [3,4], being the maximum size of these droplets is governed by a critical Webber number or ratio of the inertial forces exerted by the gas flow on the droplets, which depends on the relative velocity between the gas and the droplets, and the cohesion forces of the droplet which depend on the surface tension [3,4].

The annular flow is characterized by the presence of several types of waves at the interface between the phases, which enhance the heat transfer between the phases. The most important ones are the disturbance waves (DW) and the ripple waves (RW) [6,8]. The first ones, DWs, have a larger amplitude compared to the average thickness of the film (up to five times), they are periodic and show coherent behavior through all the sections of the annular ring of the tube, while the RWs are not periodic, and they do not show coherent behavior through the pipe section as the DWs [6]. The main characteristics of the annular flow being measured are the film thickness, the amplitude of the waves and their frequency.

Among the main methods used at present times that perform localized measurements at a given space position to obtain the time evolution are the electrical and optical procedures. These methods can give details on interfacial phenomena over remarkable time scales. Their extended use is due to their no-trouble implementation and utilization for many different applications. Employment of these techniques facilitates obtaining reasonably localized measurements, with good spatial resolutions generally of the order of millimeters. Sometimes the measurement position can be changed by displacing the probe or replicating the measurement system at different positions in the facility [9,10,11,12]. Other methods called global allow us to obtain the spatial evolution of the system at a given region of space and at different time instants for instance the wire-mesh sensor [13].

Among the most popular localized-time evolution methods that produce a minimum distortion on the film characteristics to describe the annular flow properties are the electric methods. The most popular are the capacitance methods and the conductance ones. The first one uses capacitance probes. This technique relies on the difference between the dielectric permittivity of the liquid and the gas phases, so the capacitance value obtained by placing two electrodes will be bigger or smaller relative to the thickness of the liquid layer [14].

**Figure 1:**
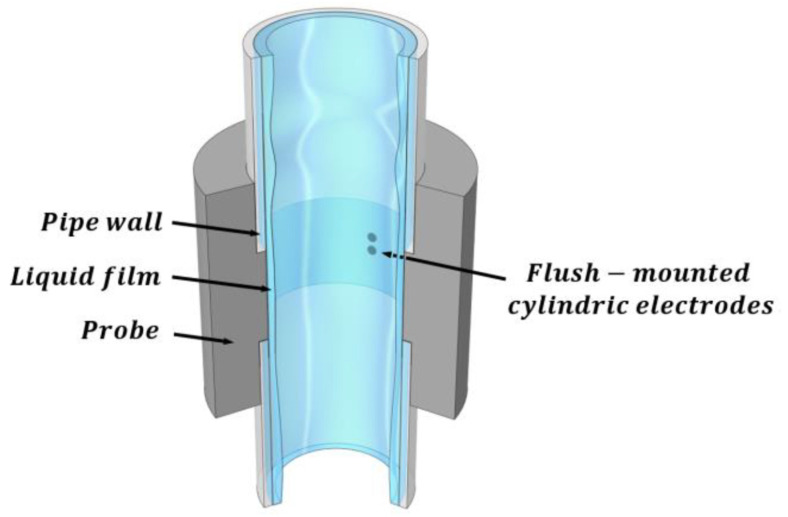
Typical mounting of the conductance probe in the test section.

**Figure 2:**
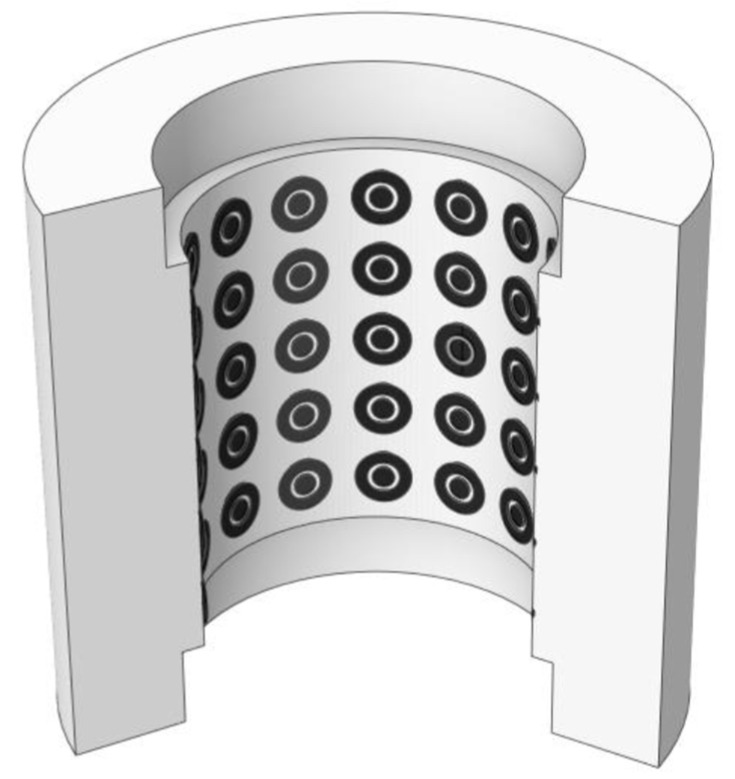
Schematic of the layout of a conductance multi-probe system.

The conductance probes are based on the difference in electrical conductance of the liquid and gas phases [15,16]. There are different types of conductance probes depending on the application, such as, for instance, the two-ring electrodes formed by two ring shape electrodes, which are mounted along the circumference of the pipe perpendicularly to the flow direction, which has been studied by Fossa [17] and Tsochatzidis et al. [18], but this type of sensor is not appropriate to perform localized measurements of the film thickness because it provides an average value over the full ring. The two-plate electrode or the two circular electrode probes are more appropriate to perform localized measurements at a given point. In addition, normally people use a third electrode connected to the earth to diminish the parasitic currents [19]. Usually, researchers apply a high-frequency alternating current (AC) to the emitter electrode to avoid high gradients of ions and redox electrochemical reactions in the electrodes, which will degrade them. As with capacitance probes, one needs to calibrate the devices considering the configuration of the electrodes to obtain the existing relationship between water conductance and film thickness. The most representative arrangement is to place the electrodes flush-mounted with the wall’s existing different configurations of the electrodes, as previously discussed, being the most popular for localized measurements the one displayed in Figure 1. Ambrosini et al. [20] were the first ones to use the conductance probe, where a two-electrode emitter and receiver are mounted flush to the flow to minimize the interference with the flow behavior and especially with the liquid film. There are also conductance multi-probe systems that use a set of small conductance probes, usually flush-mounted, that are arranged as a set of concentric electrodes as displayed in Figure 2. These electrodes are mounted on a flexible PCB (*printed circuit board*) capable of taking the same shape through which the liquid layer displaces. Each electrode couple can give a spatial resolution for the measurement of the order of millimeters [21]. The main drawback is that we can have electromagnetic coupling among the different probes.

Recently, Liu et al. [22] used a multi-probe system consisting of 160 measurement points mounted on PCB. In this study, the authors performed a comparison between their results and the results of all available empirical correlations for average liquid film thickness, base thickness (not influenced by the interfacial waves) and interfacial friction factor with very good results.

The other set of methods that allow us to obtain localized and spatial distributed results and their evolution with time are the optical methods. These methods generally use high-speed advanced cameras; the most advanced ones have high spatial resolution and can perform a high number of frames per second to obtain the thickness of the liquid film in annular flow. These methods emerge as an evolution of localized techniques and attain at present times a high level of development. The use of optical methods is the most common one due to their simplicity compared with other kinds of methods and they have the additional advantage of providing a large amount of information in their measurements. However, when applied to annular flow they present some drawbacks, as the distortion caused by light total reflection at the irregular interface between the two phases that are not uniform and contain many waves. In addition, the pipe wall distorts the film images. Xue et al. [10,11,12] and Alekseenko et al. [23,24,25] tried to correct these drawbacks using different methods based on laser-induced fluorescence (LIF). In this method, the excited species by the laser beam light will after only a few nanoseconds to microseconds, de-excite and emit light at a wavelength larger than the excitation wavelength. The most relevant optical techniques discussed are light absorption by photography, brightness-based laser-induced fluorescence (BBLIF) [26], planar laser-induced fluorescence (PLIF) [27] and some recent variants of the latter such as the PLIF40 and PLIF70 [10,11,12].

**Figure 3:**
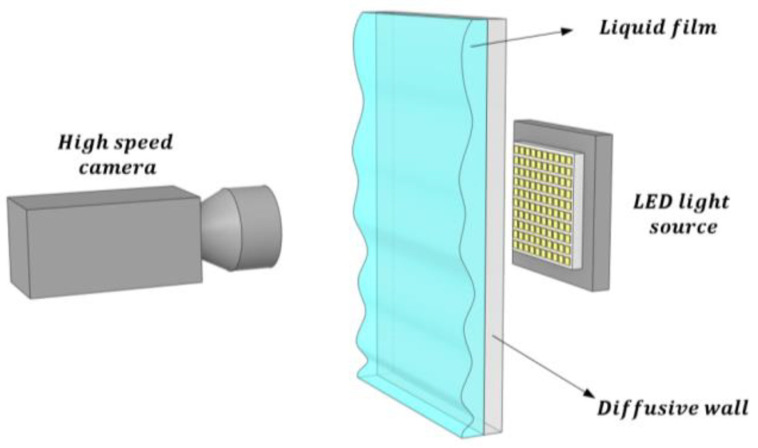
Schematic view of the main elements of the optical light absorption method.

The technique of light absorption by photography involves obtaining the quantity of light coming from a light LED source that is attenuated by the liquid layer and the wall as displayed in Figure 3. This result is obtained by using the Lambert–Beer law, which relates these two variables. In this technique, one needs to find the light attenuation coefficient of the corresponding liquid layer by calibration and, additionally, the reference intensity of the light source.

Within the optical method, one of the most used at present times is laser-induced fluorescence (LIF). This method and its different techniques are very widespread nowadays. To perform the measurements with this technique, it is required to dye the liquid phase with a fluorescent substance, commonly Rhodamine B or 6G, which is then illuminated by a laser plane beam in the measurement region. The Rhodamine B is a fluorescent organic compound with an excitation wavelength of $\lambda_{exc}=$ 553 nm and an emission wavelength of $\lambda_{emiss}=627\;\mathrm{nm}$. Otherwise, the Rhodamine 6G is also fluorescent with an excitation wavelength of $\lambda_{exc}=530\;\mathrm{nm}$ near the second harmonic of Nd—YAG laser located at 532 nm and with an emission wavelength of $\lambda_{emiss}=$ 556 nm. The key point of this technique is the wavelength difference Δλ between the laser incoming light and the emitted light by the fluorescence particles. Then, by employing a filter at the digital high-speed camera that eliminates the laser wavelengths, it is possible to remove the light rays entering the camera sensor and coming from the laser source, obtaining in this way very clean images of the liquid film [27].

**Figure 4:**
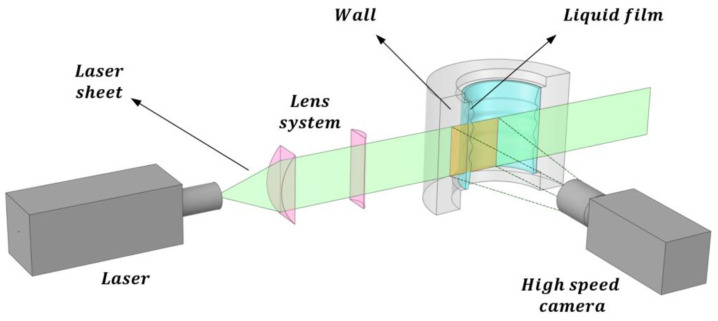
Schematic view of the PLIF technique to visualize the liquid film and the laser sheet created by the lens system.

Subsequently, a high-speed camera is employed to capture images of the liquid film. This water layer is discernible by the fluorescence emitted by the contained dye. The technique planar laser-induced fluorescence (PLIF) utilizes a planar sheet beam created by a series of lenses, generating a green-colored laser sheet as illustrated in Figure 4. Only the light emitted by the fluorescent particles reaches the camera. To mitigate the refraction of light rays emitted by the liquid layer, it is usually required for the pipe wall to possess the same refractive index as the liquid. As a common practice, a liquid containment box with flat walls is positioned around the pipe, filled with the same liquid as that flowing inside the pipe. This setup serves to prevent refraction between the outer wall and the ambient environment where the camera is situated.

Zadrazil et al. [28,29,30] use the PLIF method to make a map of all existing annular flow regimes over a large range of gas and liquid Reynolds numbers, $0\leq Re_{g}\leq 84,600$, $306\leq Re_{l}\leq 1532$ considering the characteristics of the interfacial waves. They identified four different flow regimes, namely the ‘dual wave’, ‘thick ripple’, ‘disturbance wave’ and ‘regular wave’ regimes, based on qualitative information followed by quantitative analysis, which provided information on the film thickness, interface and wave statistics, and the gas entrainment into the liquid film. The mean film thickness data obtained in these experiments were also in good agreement with previous studies. In addition, they include several kinds of measurements such as the average film thickness, roughness, frequency, amplitude of the waves, bubble frequency, and so on. They also measured the power spectral density PSD, which was obtained from temporal film thickness time traces. These authors observed that the functional relationship of the peak power frequency $f_{PSD}$ of the PSD with the $Re_{g}$ is stronger than that with the $Re_{l}$ suggesting that the gas phase has a strong role in influencing the interfacial structures and the wave dynamics. Specifically, these authors found that the peak frequency $f_{PSD}$ increases strongly with the gas Reynolds number $Re_{g}$. Also, they found that the highest frequencies were shown in the regular flow regime, and the lowest ones in the thick ripple and disturbance wave regimes.

In addition to the previous findings, Zadrazil et al. [28,29,30] also found that the PSD peak frequency $f_{PSD\;}$ correspond more closely to the more frequent, but smaller amplitude waves (i.e., ripples), rather than to the larger amplitude (e.g., disturbance) waves. They pointed out that these small-amplitude waves (RW) carry the highest amount of energy in the shape of the liquid film interface, contrary to the large-amplitude disturbance waves (DW) as many authors assumed previously.

**Figure 5:**
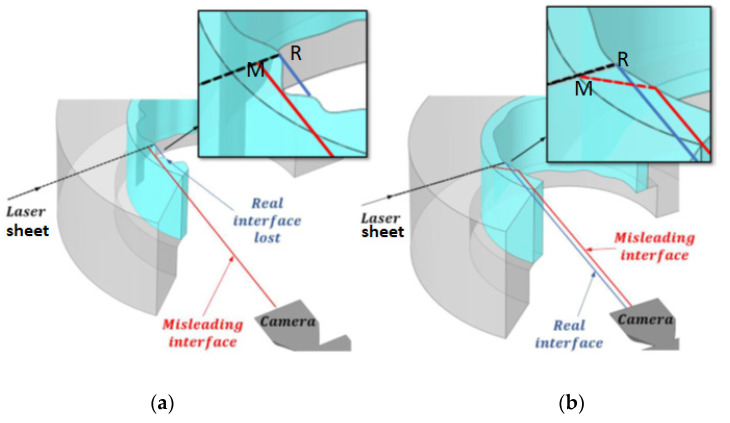
Error types occurring in measurements using LIF, (**a**) non-coherent interface error and (**b**) total reflection error at the interface.

As with the rest of the methods previously discussed, LIF has also some inconveniences. The main disadvantage that is common to all optical methods is the error produced by the light refraction as it passes from one medium to another. The annular flow is characterized by having two phases contained generally inside a curved pipe. The main error types occurring because of the refraction phenomenon have been explained by Charogiannis et al. [31] and are displayed in Figure 5.

The error source displayed in Figure 5a leads to the measurement of a smaller film thickness due to the refraction at a circumferentially non-uniform film. In this case, the real interface at R is not observed and we observe the misleading interface M, this fact leads to the measurement of a smaller film thickness than the real one (ray coming from R). This error arises due to the lack of complete coherency in the liquid layer across its entire circumferential length. In such instances, the emitted light from the interface’s fluorescent particles may interact with another section of the interface, leading to an observed film thickness that is smaller than the actual measurement. The other type of error displayed in Figure 5b may occur even if we have a circumferentially uniform film thickness and it is produced by the total internal reflection of the ray from M (red line) at the interface (between the two phases liquid and gas); we observe in this case a thicker film thickness.

**Figure 6:**
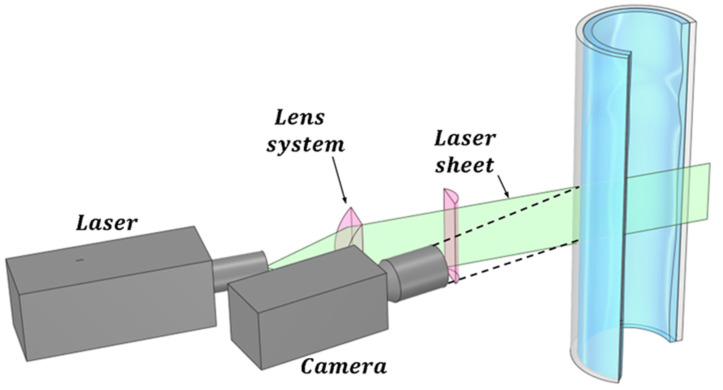
Application of the 3D-BBLIF method in pipes based on the research of Isaenkov et al. [6] and Aleeksenko et al. [25].

To avoid these errors, the LIF method has been ameliorated in recent years by two different approaches. The first one of these procedures is the brightness-based laser-induced fluorescence (BBLIF) technique developed by Alekseenko et al. [25], which is based on measuring the brightness of the fluorescent light emitted by the dye in the liquid and then converting this local brightness I(x,y,t) into film thickness h(x,y,t), considering the following relationship between both magnitudes [26]:

(1) $$I\left(x,y,t\right)=C\left(x,y\right)\left[1-e^{-\alpha h\left(x,y,t\right)}\right]\left[1+Ke^{-\alpha h\left(x,y,t\right)}\right]+D\left(x,y\right)$$

with *x* and *y* being the longitudinal and transverse coordinates, α the absorption coefficient of the fluorescent light in the dye, *K* the interfacial reflection index between phases, equal to 0.02 for moderate interface slopes, and, finally, C(x, y) is a compensation matrix created to compensate for the non-uniformity of laser illumination and to create a reference value of brightness corresponding to a reference value of film thickness. Figure 6 displays the application of BBLIF methods in cylindrical pipes as performed by Isaenkov et al. [26] in a region of interest (ROI) of 80×12 mm for cylindrical pipes. Also, Isaenkov et al. [26] performed measurements in rectangular ducts with a region of interest (ROI) of 200×50 mm. Based on these measurements, the authors concluded that the BBLIF technique can be used to measure film thickness in flat regions, but its sensitivity is smaller for thicker regions. Also, this method has some vulnerability due to light reflections in complex or agitated flows.

**Figure 7:**
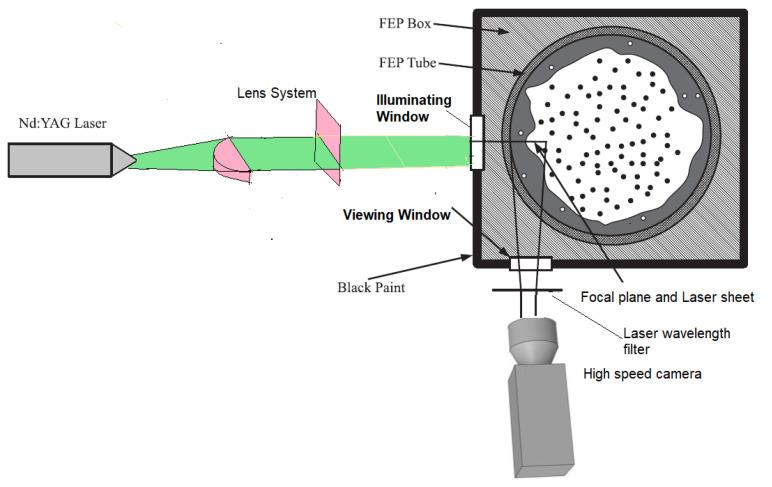
Test section for PLIF measurements with high-speed CCD camera.

The second improvement to the LIF method is the planar laser-induced fluorescence (PLIF) method displayed in Figure 4, which is widely used to investigate annular flow, but it is affected by the pipe wall and complex interfacial waves. Usually, the distortions caused by total reflection at the interface in PLIF imaging for annular flow are corrected by optical path analysis based on the assumption that the liquid film is smooth and uniform, but the interface presents different types of waves DW and RW that produce big fluctuations in the film thickness. Schubring et al. developed in 2010 [27,32] the PLIF method that consists of introducing a small concentration of a fluorescent dye (normally some type of Rhodamine) in the water, which causes the liquid film to appear as a brilliant region when exposed to laser light with the excitation wavelength of the Rhodamine. These images are then processed to locate the edge of the bright region, by application of some algorithm of edge detection, and this edge is assumed to be a gas–liquid interface. Therefore, the presence of brightness due to rhodamine emission indicates the presence of liquid film and its absence indicates the gas core of annular flow; thus, the height of the brightness region can be used with some corrections to obtain the film thickness. To minimize the effect of the refraction in the tube walls on the measurements, the authors enclose the test section into a water box with planar walls and use a round tube made of fluorinated propylene–ethylene (FEP) with the same index of refraction as the water. Two remarks about the experiment performed by Schubring [27] should be:specified the first is that the enclosure was painted in black to minimize the background light, and the second is that the laser and the camera were placed forming an angle of 90° as displayed at Figure 7, the plane of the laser sheet contained the tube axis, so the observers can view a transversal cross-section of the water film. In addition, the camera was focused on the laser sheet with a high accurate focus of 10 μm, to ensure optimal edge detection.

Recently Xue et al. [10,11,12] have proposed several improvements to obtain film thickness in PLIF measurements. The most recent improvement proposed is the PLIF40 method, which minimizes the influence of total reflection by reducing the measurement angle between the laser and the camera to 40°. In addition, they developed two methods to accurately identify liquid films with missing edge details, the first one is a sub-pixel-based edge detection algorithm and the second one is a liquid film smoothing procedure. These refinements resulted according to the authors in a measurement error of 0.030 ± 0.298 mm.

This paper compares two measurement methods, one electric (the conductance probe method with three electrodes: the emitter, receiver, and the electrode connected to ground) and one optic (PLIF40 method with subpixel correction), used to measure the film thickness in the GEPELON and CAPELON facilities located at the thermal-hydraulics laboratory of the IEE institute at the Technical University of Valencia (UPV), the first one was used to perform the comparative measurements, while the second one was used to develop the method for optical measurements.

The paper organization is as follows, Section 2 describes the GEPELON facility, the instrumentation of the facility, the data acquisition system, the instrumentation used for the conductance probes, the calibration of the conductance probes, the instrumentation used for the PLIF measurements, and the method and corrections used in the PLIF measurements. Section 3 shows the results obtained with both methods conductance and PLIF for different boundary conditions and different averaging procedures. Also, in Section 3 we discuss the results obtained with both methods analyzing the differences and trying to explain the reason for the differences. Finally, Section 4 studies the advantages and drawbacks of each method for the studied cases.

## 2. Instrumentation Used in GEPELON Facility and Methodology for Conductance Probe Measurements and PLIF Measurements

In this section, we first explain the characteristics of the GEPELON facility used to perform both the conductance probe measurements and the optical measurements with all the instrumentation used to measure the mass flow rates of gas and liquid, the pressure and the temperature, respectively. In addition, we describe all the instrumentation used to perform both the conductance probe measurements and the PLIF optical measurements. In the case of the PLIF optical measurements, we analyze the optical corrections by refraction and reflection that are necessary to achieve the correct value of the thickness, the subpixel correction of the interfacial edge [12,33,34], and the angle between the laser and the CMOS camera that is necessary to minimize the errors produced by the refraction and the total reflection.

### 2.1. The GEPELON Facility and Its Instrumentation

The GEPELON experimental flow facility (GEneración de PELícula ONdulatoria or translate to English Wavy Film Generation) is an installation designed to generate an annular two-phase flow. This equipment has been designed to generate a layer of liquid sliding on the pipe walls while the air mass remains in the core part of the pipe, either quiescent or moving in co-current flow, i.e., GEPELON produces a downward annular air-liquid flow moving downward through the vertical test section of the facility. The expected phenomena to occur will be like the ones of the passive cooling systems in third-generation reactors or small modular reactors (SMR), or like the ones occurring in steam generator tubes during some accidental scenarios, but only in their hydraulic performance. In this phase of the facility, there is no incorporation of heated fluids, meaning that it does not account for heat transfer between the fluids or the wall. However, it should be noted that a recent upgrade has been implemented to enable the system to accommodate heated fluids. In the present study, we will display the results obtained with water as fluid at three temperatures 20 °C, 30 °C and 40 °C, the temperature control system of GEPELON allows us to control the temperature in the circuit with very high precision. 

**Figure 8:**
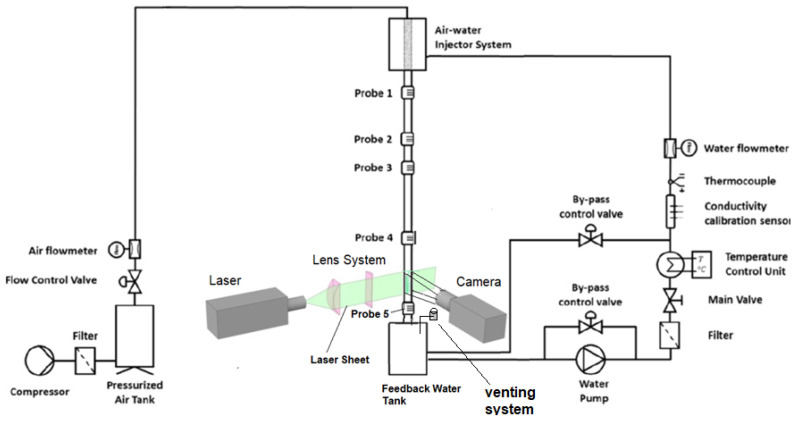
Layout of the GEPELON facility with the conductance probes and the PLIF measuring system.

The facility configuration is displayed in Figure 8 and has been previously summarized by Rivera et al. [9]. In this case, the figure shown is slightly different from previous ones because we have added the instrumentation necessary to perform the PLIF measurements. The operative length of the experimental test section is approximately 3.8 m. The setup comprises two distinct test segments, with only one of them illustrated in the accompanying figure. These test segments consist of two pipes with internal diameters measuring 30 mm and 42 mm, respectively. The facility is equipped with two separate circuits, one for air and the other for water. This arrangement allows for the independent preparation of air and water properties to meet specific conditions before their introduction into the test section. Then, the facility is formed by the following set of components: the air pumping system, the water pumping system, the injection/mixing system, the test section and the water collection/recirculation system. This group of components is equipped with various devices and sensors, thus being able to measure and/or control the main variables necessary to know/modify the experimental conditions and to be able to perform the subsequent analysis.

The water used in all experimental data sets is carefully managed before being supplied to the loop. Its properties are adequately prepared using filters, temperature controller and PH controller to have the appropriate conductivity without impurities.

Along its path from the feed water tank to the injector, the liquid goes through several devices to control and measure its properties. In particular, the conductivity calibration sensor, displayed in Figure 8, records any variation in the water conductivity before performing the measurements. The recorded values are transmitted to the data acquisition program (DAQ) for the case-dependent calibration of the probes. Nevertheless, due to the limited temperature variability maintained by the temperature control unit, the conductivity experiences minimal fluctuations once the facility has reached a state of operational stability. Furthermore, a pump inverter, which monitors the water flow rate and employs a PID controller, sustains the pump power at the desired operational point. In addition, a temperature control station maintains the water temperature in the circuit at the desired temperature value, performing the necessary changes to keep it constant. Numerous control and safety valves, filters, and other devices have been installed, as illustrated in Figure 8, within the circuit to serve distinct functional objectives.

In the free-fall configuration, a water pump, capable of exerting a maximum pumping pressure of 4.2 bar, moves the water along the system close loop. The pump conveys the water to the upper section of the standpipe via a custom-designed pressurized water injection system [35]. Being the mass flow rate of liquid injected by this device proportional to the pressure difference between the two sides (outer and inner) of the wall of the porous sintered pipe. Due to this pressure difference, the water passes from a small water reservoir to the inner wall of the sintered steel pipe, forming a water film. Then, by gravity, the water descends sliding on the inner wall of the methacrylate tube and flows downward sliding on the walls of the test section, and, finally, the water is collected in a storage tank. This water reservoir is a large tank, from which the water recirculation pump sucks the fluid and pushes it back into the small injector system tank, so that the water starts a new cycle. The pore size of the porous material of the sintered steel pipe component is $19\times 10^{-6}$ m and the coefficient of viscous permeability, $y_{S}$, is $0.8\times 10^{-12}$. The pressurized water injection system, the test section, and the separation tank, displayed in Figure 8, are doubled as previously explained.

The facility configuration for co-current experiments is the same one as for free fall experiments being the only difference in the air injection system. The air stream is filtered, de-dusted and demisted prior to its injection into the upper part of the facility, as displayed in Figure 8. The components of this circuit are the following ones, a compressor, a big stabilization tank to ensure a constant air flow rate, a gas flowmeter, and several safety and control valves. In addition, there is a draining air tube with a valve located in the upper part of the feedwater water tank. The compressor has a maximum working pressure of 8 bars and a maximum volumetric flow rate of 3750 L/min, even though only air flows up to 2500 L/min have been reached in the experiments. The test section comprises two nearly four-meter-long vertical methacrylate tubes. The liquid film characteristics are measurable through conductance probes positioned at five distinct distances from the point of water flow entry along the test section. Therefore, five ports and five conductance probes have been installed at different distances to study the development of the flow and the interfacial waves with the distance.

### 2.2. The Conductance Probes and Its Calibration

Conductance probes are based on the conductance value measured between the emitter and receiver electrodes at high frequencies, which depends on the liquid film thickness [15,16,17,19,36]. This technique is one of the most used when trying to measure temporal variations of film thicknesses because the sampling rate of the measurements can attain very high values as $10^{5}$ sample/s, providing the evolution of the waves with time with a good precision that basically depends on the separation between the center of the electrodes. Also, this technique is cheaper than the LIF technique, as will be discussed later. The operation of conductance probes adheres to the principles of potential field theory. Under this framework, the response of the liquid layer located between the electrodes, when subjected to an alternating current, exhibits resistive behavior. This behavior is attributed to the notably low electric permittivity, and the frequency-dependent behavior becomes marginal at elevated frequencies [15].

The conductance probes work because there is a proportionality between the liquid layer thickness located between the electrodes and the current transmitted between them; the reason is that the conductivity of the liquid is much higher than the gas conductivity. The conductance probe comprises three electrodes that are integrated into the wall, positioned in parallel or perpendicular alignment to the direction of fluid flow. The emitting electrode, situated at one extremity of the device, sends a sinusoidal signal with a frequency of 100 kHz and an amplitude of 5 Vpp coming from a signal generator. One of the electrodes is connected to the ground normally the central one, while the position of the receiver electrode is at the other end of the device. This last electrode receives the signal and sends it to the filtering and processing device. The way the conductance probe works is as follows: the first electrode emits an electrical AC signal, which is then transmitted through the thin liquid film, and finally captured by the receiver electrode, resulting in a direct correlation between the received signal and the thickness of the liquid layer. The mission of the ground electrode is to collect the parasitic currents.

**Figure 9:**
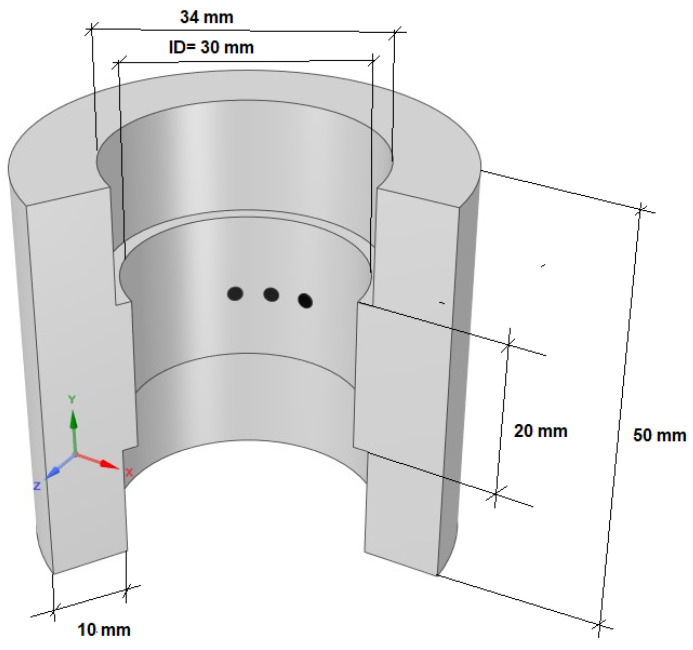
Port design for the conductance probe measurements of these experiments, the electrodes have a diameter of 1.5 mm and the distance between electrodes is 1.5 mm. The port length is 170 mm and only a portion is shown in the figure.

The port design of the conductance probes has been built by 3D impression because it guarantees the perfect alignment of the conductance probes and the distance among them. The first ones were manufactured mechanically but this procedure was discarded because we do have not in our laboratory the necessary precision to manufacture the sensor with the required accuracy for small electrodes and the distance between them. Figure 9 shows the port design along with the probes used in these experiments, the total length of the port is 170 mm and has an internal diameter in its central part equal to the test pipe diameter, i.e., 30 mm. Each conductance probe has three 1.5 mm ID electrodes, each spaced 1.5 mm and aligned perpendicularly to the flow direction. The conductance probe where the measurements were performed is located near the lower part of the test section near the bottom test entrance to have developed flow conditions. For the 30 mm diameter pipe, this distance was 117 hydraulic diameters (351 mm). The selection of the electrode diameter and the distance between electrodes was achieved as a compromise between the desired spatial resolution of the measurements, which diminishes with the total distance between the emitter and receiver electrodes and the range of the measurements or capability to measure larger thickness, which increases with the distance and size of the electrodes.

**Figure 10:**
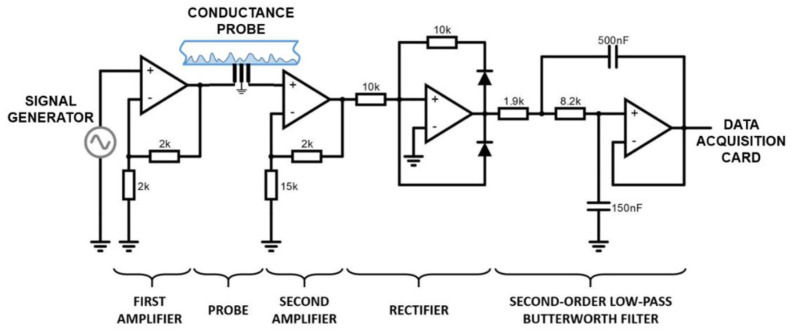
Detailed scheme of the electronic circuit associated with the conductance probes.

The electronic circuit associated with the conductance probe remains consistent with the one employed in previous publications [9]. The electronic design has the capability to emit, amplify, filter and receive the electric signal provided by the signal generator with a given frequency, and is shown in Figure 10. It comprises four high-frequency amplifiers, a pair of precision resistors (0.1% tolerance) and 1N4148 diodes. The electronic second-order low-pass filter is designed to cut off frequencies above 149 Hz using the Sallen–Key architecture [37]. Finally, a Butterworth filter was used to have an attenuation above the cut-off of −20 dB/decade.

A signal generator displayed in Figure 10 generates a high-frequency $10^{5}\;\mathrm{Hz}$ sinusoidal AC wave signal that travels to the emitting electrode. After amplification, the electric field generated by this signal travels to the receiving electrode through the water film and the electric current intensity entering the receiver electrode depends on the thickness of the water film located between both electrodes. Subsequently, the received signal undergoes amplification, rectification, and filtration to yield a direct current (DC) signal. This DC signal is ultimately acquired through a data acquisition card and stored in the computer for later post processing [9]. The units registered of the raw signal at the receiver electrode are volts and need to be converted to film thickness (mm), this stage is performed using a calibration procedure of the conductance probe that is explained below.

**Figure 11:**
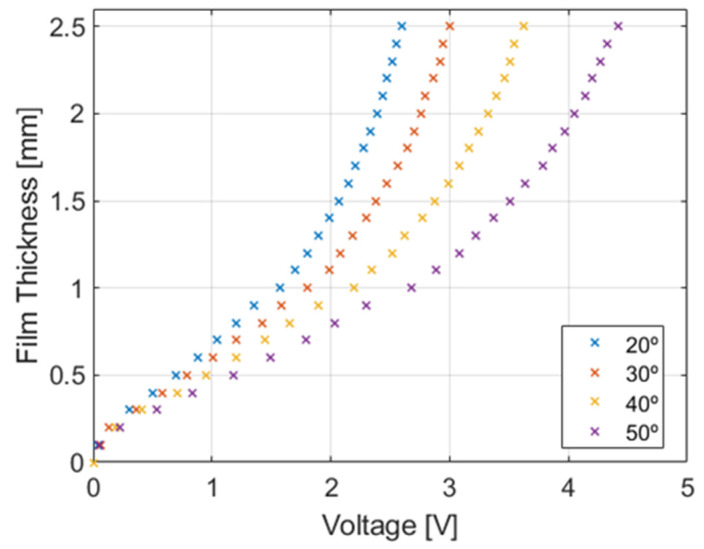
Calibration Points of the Conductance Probes up to its saturation point at 20 °C, 30 °C, 40 °C and 50 °C for the test tube with 30 mm ID.

The calibration procedure was performed in two steps: in the first step, we obtained the calibration curves at different temperatures and for several film thicknesses using the same method used by Rivera et al [8,9] to check whether the shape of the curve is of the same type, i.e., a polynomial degree of 5 order for all the temperatures; see Figure 11. Confirming this result would allow us to use only the database generated at 20 °C for calibration purposes at any temperature between 20 °C and 50 °C, simply scaling the voltage value obtained for each specific base case test at 20 °C with the saturation voltage measured prior to each experimental data set at a given temperature.

Based on this idea, the calibration voltage for a given thickness for each temperature run has been carried out by measuring the saturation voltage $V_{sat}{}^{scaled}$ at the beginning of each experimental data set, and then using this saturation value to correct the voltages of the base case. The new calibration curve has been obtained using the following interpolation expression to scale the calibration points of the base case $V_{i}{}^{b}$ at 20 °C to the new temperature conditions.

(2) $$V_{i}{}^{scaled}=V_{min}{}^{scaled}+\left(V_{i}{}^{b}-V_{min}{}^{b}\right)\cdot \frac{\left(V_{sat}{}^{scaled}-V_{min}{}^{scaled}\right)}{\left(V_{sat}{}^{b}-V_{\;min}{}^{b}\right)}\;$$

where the superscripts *b* and *scaled* denote the base calibration values and the scaled calibration values, respectively.

Finally, to finish this section, we discuss the calibration procedure performed for all conductance probes. The device employed consists of the following parts: an electronic precision positioning system; a flatness optical table; and several dielectric cylinders of different diameters. The positioning system places each cylinder of known diameter at the central position of the inner side of the conductance probe port. Then, a high-precision positioning system allows us to know the exact placement of the cylinders, so that the exterior side of the cylinder and the interior side of the probe have a constant known gap in their entire circumference. This gap is then filled with treated water. Then, the calibration procedure includes the following steps: The signal generator sends a specific signal, which excites the probe’s emitting electrode. The electrical signal passes through the known liquid layer thickness, and the correspondent signal is received by the probe’s receiving electrode. This electrical signal is then collected by the DAQ. Subsequently, the relationship between the voltage of the electrical signal and the liquid film thickness can be obtained, as the liquid thickness is known, and the signal voltage is being measured. 

This process is then replicated for all-dielectric cylinders to acquire a sufficient number of calibration points that establish the correlation between signal voltage and liquid film thickness for each probe. In addition, the GEPELON facility has a temperature control unit to maintain the temperature of the circuit at a constant fixed value. By using this system, GEPELON achieves high-temperature stability, and it is observed that the conductivity does not vary once the facility attains stable temperature conditions.

### 2.3. The Methodology Used for PLIF Optical Measurements in GEPELON Facility and the Correction of Refraction and Total Reflection Errors

As commented in the introduction section, in the method of planar laser-induced fluorescence (PLIF), a small concentration of a fluorescent dye (Rhodamine) is added to the water causing the liquid film to appear as bright regions on the images once exposed to laser light with a wavelength equal to the excitation wavelength of the rhodamine. These images are then processed to locate the edge of the bright region, asserted to be a gas–liquid interface. The main difficulty arises because there are different types of errors as total reflection at the interface and refraction in the tube wall that can cause misleading film thickness measurements. Xue, Li, and Zhang [12] recently performed two kinds of optical corrections to minimize the errors caused by refraction in the wall and total reflection at the interface.

One of the problems we found is the reflection and the refraction of the laser light in the tube walls making it very difficult to find the interface between the tube wall and the liquid. This problem is easily solved because when the laser photons interact with the dye, the wavelength changes from 532 nm to 610 nm. This means that if we place an optical filter in front of the camera lens, we can remove the laser’s base wavelength and keep only the wavelength of interest. In theory, this would allow us to observe only the liquid, completely solving one of the problems: determining the location of the inner tube wall.

**Figure 12:**
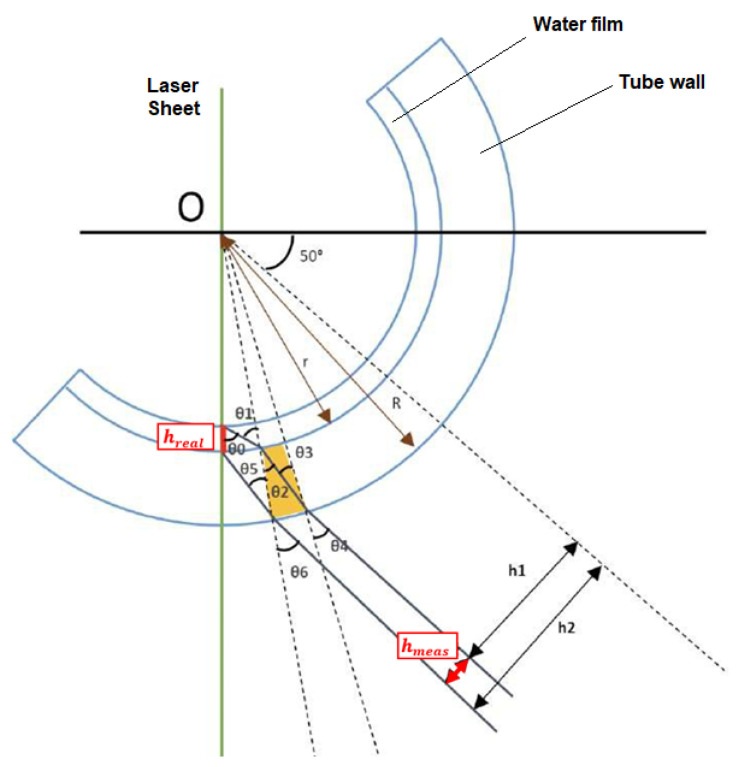
Diagram of the main variables to calculate relation between real and measured film thicknesses (the laser sheet is the green line).

In addition, to mitigate the challenges posed by refraction when measuring annular flow within a pipe, many researchers employ the liquid box technique [27]. This technique requires a carefully designed configuration involving the use of a specific tube with the same refractive index as the fluid under investigation (like FEP or PFA for water at ambient temperature measurements). Furthermore, this tube needs to be enclosed within a box filled with the same fluid under study, creating a refractive index-controlled environment. In this setup, the photons coming to the camera from the region of interest (ROI) pass through media with similar refractive indices until they encounter the box’s walls, which are positioned perpendicular to the camera’s field of view as displayed in Figure 7. However, it is possible to bypass this configuration if the refractive index of each medium is known. To achieve this, the actual film thickness as perceived by the camera can be calculated using the following methodology. Figure 12 shows a schematic diagram illustrating the different angles and distances used for this correction, which are needed to obtain the true film thickness $h_{r}$ from the measured film thickness $h_{meas}$.

Figure 12 depicts a cross-sectional view of the tube, denoted by the two outermost arcs, and the liquid film within it, represented by the innermost arc. The central point of the tube is designated as O. The green line in the diagram indicates the laser sheet, and the camera is positioned at a fixed angle of 40 degrees (PLIF40). The true thickness of the liquid film is denoted as $h_{real}$ or $h_{r}$, while the apparent measured thickness is represented as $h_{meas}$. Additionally, the inner radius and outer radius of the tube are denoted as r and R, respectively. The corrections needed were programmed in Matlab 2021 (MathWorks, Natick, MA, USA), and the main steps of the program are explained next.

To establish a meaningful relationship between $h_{meas}$ and $h_{r}$, it becomes necessary to compute all the angles that play a pivotal role in the refraction process. According to Snell’s law, we can formulate the following equations to describe these angles:

(3) $$n_{w}\sin\theta_{1}=n_{p}\sin\theta_{2}$$

(4) $$n_{p}\sin\theta_{3}=n_{a}\sin\theta_{4}$$

(5) $$n_{p}\sin\theta_{5}=n_{a}\sin\theta_{6}$$

Here, $n_{w}$ represents the refractive index of water, $n_{p}$ the refractive index of the tube, and $n_{a}$ the refractive index of air with values 1.33, 1.49 and 1, respectively. Additionally, the angle $\theta_{0}$ can be expressed based on the angles $\theta_{1}$ to $\theta_{4}$ and the position of the camera at 40° following the next relation:

(6) $$\theta_{0}=40{}^{\circ}+\theta_{1}-\theta_{2}+\theta_{3}-\theta_{4}$$

**Figure 13:**
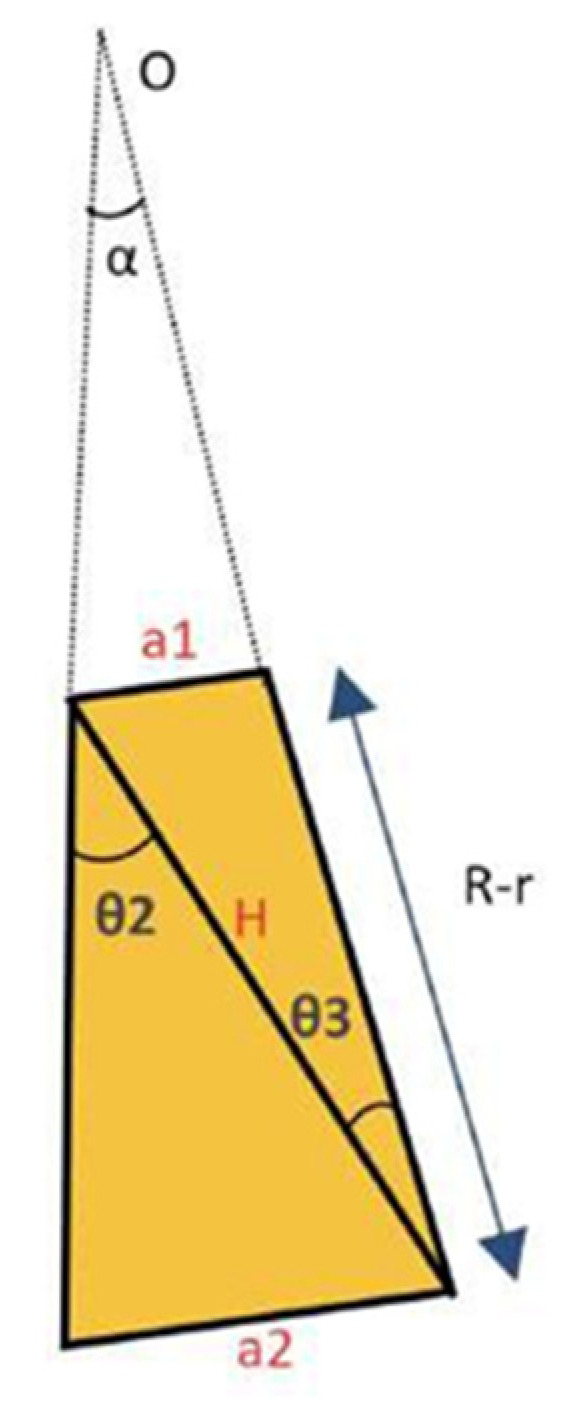
Schematic diagram of the relation between angles.

To relate all the angles, we need to consider their relationships as shown in Figure 13. Then, from Figure 13, it is obtained:

(7) $$a_{1}=2r\sin\left(\frac{\alpha }{2}\right)$$

(8) $$a_{2}=2R\sin\left(\frac{\alpha }{2}\right)\;$$

Consequently, the angles $\theta_{2}$ and $\theta_{3}$ can be determined as follows:

(9) $$\sin\left(\theta_{2}\right)≅\;\frac{a_{2}}{H}=\frac{2R\sin\left(\frac{\alpha }{2}\right)}{H}$$

(10) $$\sin\left(\theta_{3}\right)\;≅\;\frac{a_{1}}{H}=\frac{2r\sin\left(\frac{\alpha }{2}\right)}{H}$$

From Equations (9) and (10), it follows:

(11) $$\frac{r}{\sin\left(\theta_{3}\right)}=\frac{R}{\sin\left(\theta_{2}\right)}$$

It is possible to generalize the previous expression to the entire system:

(12) $$\frac{r-h_{real}}{\sin\left(\theta_{1}\right)}=\frac{r}{\sin\left(\theta_{0}\right)}$$

Similarly, as carried out for angles $\theta_{2}$ and $\theta_{3}$, it is possible to determine the relation between $\theta_{5}$ and $\theta_{6}$:

(13) $$\frac{r}{\sin\left(\theta_{5}\right)}=\frac{R}{\sin\left(40{}^{\circ}-\theta_{6}+\theta_{5}\right)}$$

The only missing term gives the relation of the angles $\theta_{4}$ and $\theta_{5}$ with the measured thickness $h_{meas}$, which can be obtained from $h_{2}$ and $h_{1}$ distances, depicted in Figure 12 as follows:

(14) $$h_{meas}=h_{2}-h_{1}=R\sin\left(\theta_{6}\right)-R\sin\left(\theta_{4}\right)=R(\sin\left(\theta_{6}\right)-\sin\left(\theta_{4}\right))$$

With this set of equations, it becomes possible to iterate and derive the true film thickness value ($h_{real}$). Upon manipulating the set of equations derived previously, the resulting system is as follows:

(15) $$R=\frac{r\;\sin\left(40{}^{\circ}-\theta_{6}+\theta_{5}\right)}{\sin\left(\theta_{5}\right)}$$

(16) $$\sin\left(\theta_{1}\right)=\frac{R}{r}\frac{n_{a}}{n_{w}}\left(\sin\left(\theta_{6}\right)-\frac{h_{meas}}{R}\right)$$

(17) $$\sin\left(\theta_{0}\right)=\sin(40{}^{\circ}+sin^{-1}\left(\frac{R}{r}\frac{n_{a}}{n_{w}}\left(\sin\left(\theta_{6}\right)-\frac{h_{meas}}{R}\right)\right)-sin^{-1}\left(\frac{R}{r}\frac{n_{a}}{n_{p}}\left(\sin\left(\theta_{6}\right)-\frac{h_{meas}}{R}\right)\right)\;+sin^{-1}\left(\frac{n_{a}}{n_{p}}\left(\sin\left(\theta_{6}\right)-\frac{h_{meas}}{R}\right)\right)-sin^{-1}\left(\sin\left(\theta_{6}\right)-\frac{h_{meas}}{R}\right)$$

(18) $$h_{real}=r\left(1-\frac{\sin\left(\theta_{1}\right)}{\sin\left(\theta_{0}\right)}\right)$$

As mentioned before, this system of equations requires an iterative approach for resolution. To speed up the correction process to obtain the true value of the film thickness, it is possible to establish a fitting relationship between measurements and real value using a straightforward polynomial equation of second degree as has been carried out by Xue et al. [12]. For this set of experiments, the polynomial equation that relates the measured and true thickness is:

(19) $$h_{real}=-0.0415\;h_{meas}{}^{2}+1.9753\;h_{meas}-0.0014$$

The effect of the temperature on the refraction index of the water is very small around $10^{-4}$/°C, so for a variation of 30 degrees Celsius, the effect of the temperature on the refractive index can be neglected.

**Figure 14:**
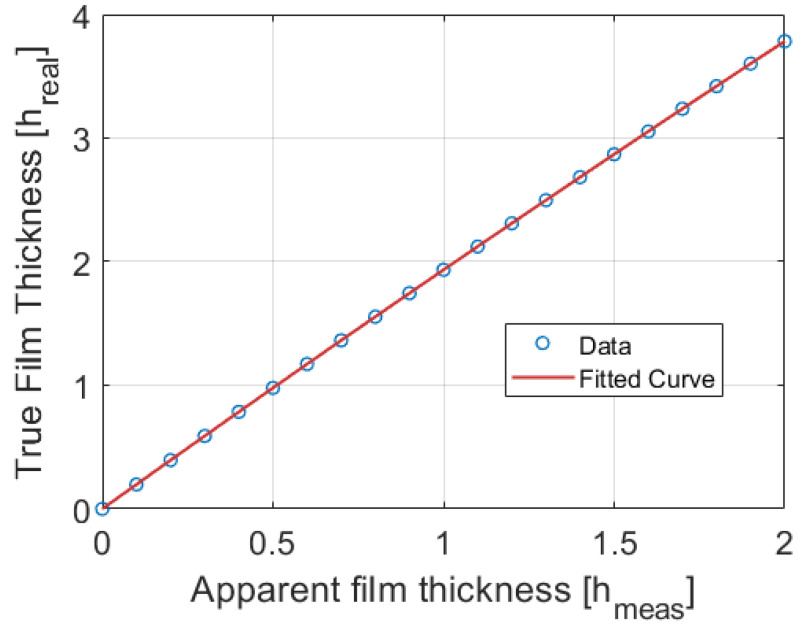
True film thickness values computed with the correction algorithm versus measured values and fitting curve results of Equation (19).

Equation (19) was obtained by fitting the data of the true values computed with the algorithm with a polynomial function in terms of the measured values. The determination coefficient was practically 1 and the data and the fit curve are displayed in Figure 14.

### 2.4. Images Processing and Subpixel-Edge Detection in the PLIF40 Optical Measurements

**Figure 15:**
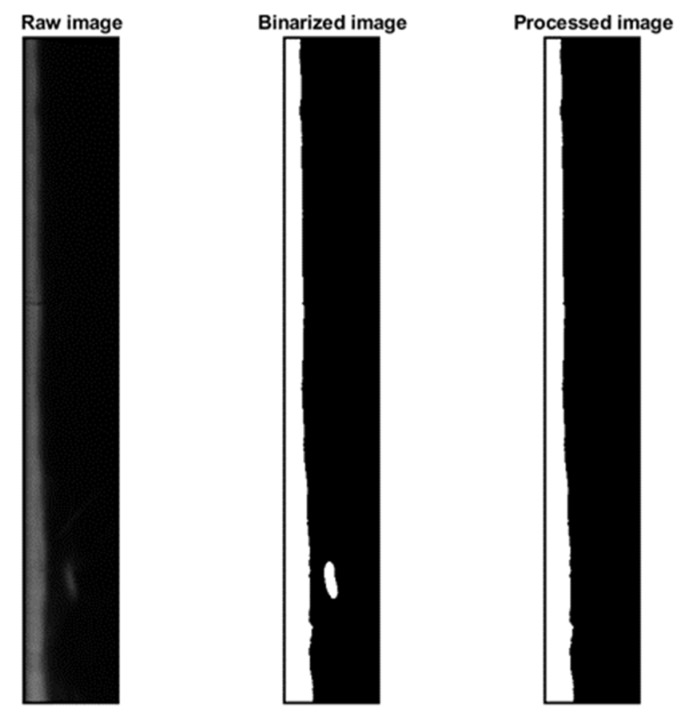
Comparison of the grey image of the water film edge with its binarized image.

Based on the experiments of previous authors [12] and after checking it in the laboratory we decided to use the PLIF40 measurements. finding an efficient algorithm that meets the specifications. First, let us focus on a single image. The format of the saved images is TIFF, and to read this format in a Matlab image processing kit, the images are read as 3-dimensional matrices, which is not ideal for manipulation. Therefore, we use the “rgb2gray“ function of Matlab to convert the colors to grayscale (values ranging from 0 to 255), which gives us 2-dimensional matrices. First, we noticed that the difference between the two images is very small and practically negligible. To convert our grayscale image to a black and white image, we need to determine a threshold. Once the threshold is set, we compare each cell with this threshold: if the value in the cell is less than the threshold, the pixel is black; otherwise, it is white. To determine the appropriate threshold, we calculate the average of all values stored in the cells and this value will be used as threshold. After coding this procedure and processing the images, the result is satisfactory, and it is possible to clearly distinguish the liquid because some perturbing refractions that appear in the images have been removed, as displayed in Figure 15. Then, the function “bwperim” of Matlab is applied to the binarized image that returns a binary image containing only the perimeter pixels (contour) of objects in the input image. But this method produces errors when we have bubbles in the liquid film layer or when the interfacial waves fold back. In these cases, the measured film thickness is distorted and some kind of correction becomes necessary. The normally applied algorithm to correct these situations is the sub-pixel detection algorithm.

Sub-pixel detection is a widely used method for object detection and computer vision. This method is much more accurate than pixelated contour detection (the method used previously). For this study, we will use a detection operator called Sobel operator used in artificial vision for edge detection [33,34]. Sobel filtering involves applying two 3 × 3 convolutional kernels or filters denoted as $G_{x}$ and $G_{y}$ to an image. These two kernels detect the edges in the image in the horizontal and vertical directions. They are applied separately and then combined to produce a pixel value in the output image at each position in the input image. The two filters used by this operator are defined as follows. If I denote the image to be transformed, then the Sobel filter acts in two steps:

Horizontal changes. First, it performs the convolution of the image with the horizontal Sobel kernel, i.e., it performs the following operation:

(20) $$G_{x}=\left[\begin{array}{ccc} -1 & 0 & 1\\ -2 & 0 & 2\\ -1 & 0 & 1 \end{array}\right]*Image$$

Vertical changes. Second, it performs the convolution of the image with the vertical Sobel kernel, i.e., it performs the operation:

(21) $$G_{y}=\left[\begin{array}{ccc} 1 & 2 & 1\\ 0 & 0 & 0\\ -1 & -2 & -1 \end{array}\right]*Image$$

If f(x=i,y=j) denote the matrix elements of the image, then the result of the step 1 and 2 on the image produce the following elements.

Horizontal filter operator of Sobel:

(22) $$G_{x}=-f\left(x-1,\;y-1\right)+f\left(x+1,\;y-1\right)-2f\left(x-1,\;y\right)+2f\left(x+1,\;y\right)-f\left(x-1,\;y+1\right)+f\left(x+1,\;y+1\right)$$

Vertical filter operator of Sobel:

(23) $$G_{y}=-f\left(x-1,\;y-1\right)-2f\left(x,\;y-1\right)-f\left(x+1,\;y-1\right)+f\left(x-1,\;y+1\right)+2f\left(x,\;y-1\right)+f\left(x+1,\;y-1\right)$$

Therefore, the operators $G_{x}$ and $G_{y}$ retrieve information about the intensity values of the neighboring pixels at 8 different locations relative to the current pixel being processed. To identify edges within an image, we perform a convolution operation on the entire image using these filters as performed in Equations (20) and (21). The result of this convolution, known as the gradient (*G*), combines the strengths of both the horizontal and vertical gradients [34]:

(24) $$G=\sqrt{G_{x}{}^{2}+G_{y}{}^{2}}$$

(25) $$\theta =tan^{-1}\left(G_{y}\;/G_{x}\right)$$

*G* represents the overall strength of the gradient at each pixel. By comparing this gradient strength to a predefined threshold, we can classify a pixel as an edge pixel, with the threshold influencing the sensitivity of the edge detection process. Additionally, we can determine the direction of edges *θ* to provide the orientation of the detected edge, distinguishing between horizontal and vertical edges. Comparing magnitudes of $G_{x}$ and $G_{y}$, it is possible to directly classify the point as a horizontal or vertical edge point as follows:

If $G_{x}\left(x,y\right)>G_{y}\left(x,y\right)$, then the point (x,y) is classified as a horizontal edge point.

If $G_{y}\left(x,y\right)$ > $G_{x}\left(x,y\right)$, then the point (x,y) is classified as a vertical edge point.

Applying the previous algorithm to each experimental snapshot, it is possible to identify the film thickness with better accuracy compared with the traditional pixelated contour detection, accurately indicating the liquid evolution.

In conclusion, the action of the Sobel operator on the image can be seen as a two-dimensional mapping of the gradient on each image point, where the regions with high gradients represent the edges and the rest is the background.

**Figure 16:**
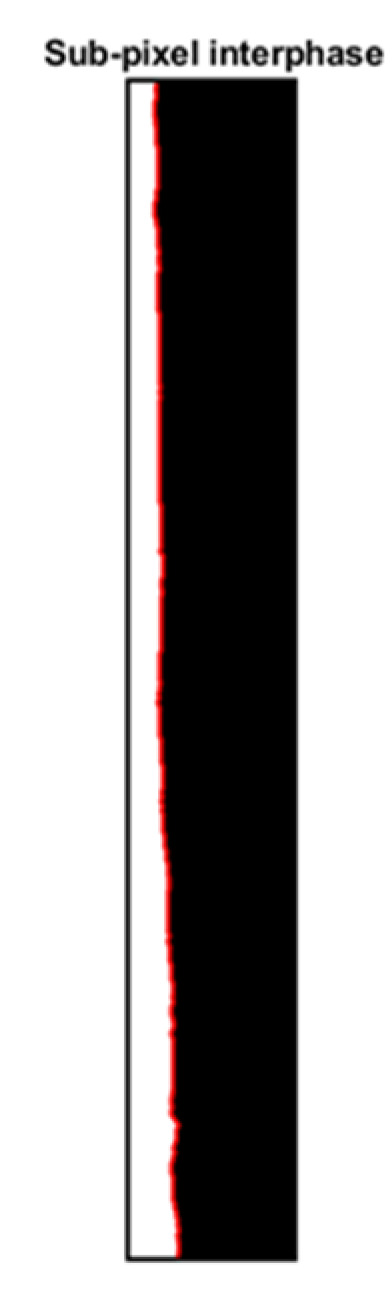
Result of the application of the subpixel detection algorithm to the grey image (layer edge in red color).

The implementation of the sub-pixel detection algorithm produced the image displayed in Figure 16, where the film layer edge is clearly distinguished.

Finally, to convert the number of pixels of the recorded images into length (mm), we locate a reference length rule with an imprinted length scale in the position of the laser sheet and we count the number of pixels located between two marks, obtaining the equivalence in mm between pixels and length. The result was that 1 mm is equivalent to 30 pixels.

Normally in the Sobel algorithm, the larger distance is denoted as the *x*-axis and the shorter distance *y*-axis. So, the *x* coordinates will be in the axial direction and the *y* coordinates in the radial direction. In our images we take the *x* coordinates in the normal direction to the pipe wall and name it as *x*′ and the *y* coordinates in the direction of the pipe axis and denote it *y*′ so we perform the change:

(26) $$G_{y^{\prime }}=G_{x}\;and\;G_{x^{\prime }}=G_{y}$$

So, the values of G do not change according to Equation (24).

**Figure 17:**
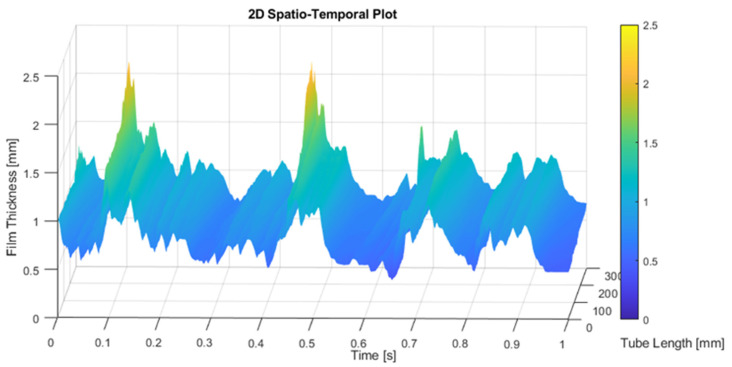
Time evolution (frame number).

**Figure 18:**
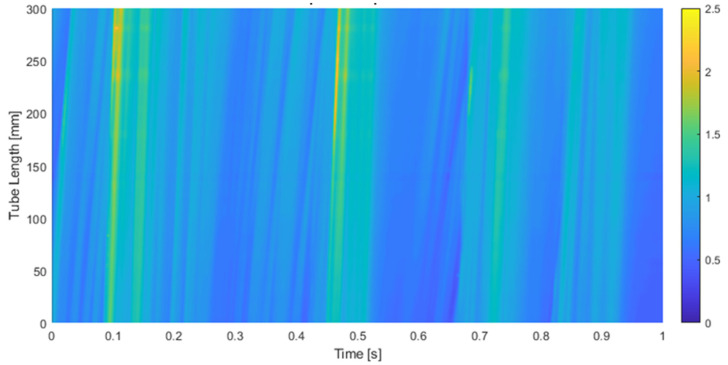
Time evolution of the waves with time. It is observed that all the DWs have approximately the same celerity.

Next, we display in Figure 17 the film thickness in mm (*z*-axis) at different positions (*y*′-axis) versus the time (frame number) for the measurements performed in annular flow with subpixel edge detection. The position along the *y*′ axis was expressed in pixels (30 pixels is 1 mm), the time was expressed in frames and the CMOS camera worked at 1940.1 Hz or frames per second. It is observed that the disturbance waves (DW) propagate all with the same celerity given by the slope of the straight lines displayed in Figure 18. The maximum of these waves is shown in yellow color while the minimum is in blue. One observes that not all the waves display the same slope, and the waves with smaller amplitude show a smaller slope.

### 2.5. Treatment of the PLIF40 Images for Determination of Temporal Film Thickness Evolution

To effectively extract the crucial characteristics defining the behavior of the film under study, the development of a rigorous signal postprocessing methodology becomes imperative. This methodology must not only ensure the accurate analysis of the film but also guarantee consistency in handling data obtained from different measurement approaches. As the primary objective of this research is to compare the outcomes derived from both the conductance probe and PLIF methodologies, it is essential to establish a methodology that harmonizes the data processing for both techniques, considering the unique aspects of each measurement approach.

For the PLIF snapshots, we have previously explained the algorithm employed to extract the interface, and, consequently, the film thickness. However, the subsequent step demands the generation of a comprehensive data set including all the images acquired during each experimental run. One notable advantage of the PLIF system relies on its capability to capture the spatiotemporal measurements, enabling the assessment of the film’s evolution from both spatial and temporal perspectives. In contrast, the conductance probe measurements offer insights solely into the temporal evolution of film thickness at a specific location within the test section. To facilitate a meaningful comparison between these two instrumentation types, we initially process the PLIF measurements to extract the temporal evolution in a similar way as the conductance probes. This involves calculating the average film thickness within a 7.5 mm window for each snapshot and aggregating these values over the entire run. The choice of a 7.5 mm window aligns with the physical separation of the probe electrodes (emitter and receiver), providing a basis for equitable comparison. At this point, both sets of measurements are rendered equivalent, and the treatment for extracting the primary film variables has been standardized across the two methodologies.

The mean film thickness, denoted as $h_{mean}$, corresponds to the overall average of the film thickness values for each experimental run. Properties associated with disturbance waves, including their height ($h_{DW}$) and frequency ($\nu_{DW}$), are estimated by identifying the main peaks within the signal. This peak detection process relies on three key parameters. Firstly, the minimum peak height is set to determine the threshold thickness for considering a peak as a disturbance wave, with this value being established as the mean film thickness, as documented in previous studies by Rivera et al. [8], ensuring the consistent identification of disturbance waves. Secondly, a minimum distance between peaks is defined to mitigate the detection of single disturbance waves as multiple waves when dispersion or detachment of droplets occurs. This parameter is set at 1/1000 times the frame rate, which is almost one hundred times lower than the expected frequency, effectively eliminating such erroneous identifications. Lastly, to enhance the algorithm’s robustness, a minimum prominence value for the peaks is set to exclude low-amplitude peaks that might correspond to minor ripple waves. A threshold of 0.2 mm is established, ensuring the exclusion of overlapping ripple waves for the cases under this study. The height of the disturbance waves, $h_{DW}$, is therefore determined by calculating the average thickness of the detected peaks, while the frequency of the disturbance waves, $\nu_{DW}$, is computed as the mean number of peaks detected divided by the recorded time duration. This comprehensive signal postprocessing methodology facilitates the extraction of key film characteristics, ensuring that the comparison between the two measurement techniques is both rigorous and meaningful.

### 2.6. Treatment of the LIF Images to Study the Spatial Evolution of the Film Thickness

As previously mentioned, the spatial evolution of the film thickness can be analyzed using PLIF measurements. This method involves detecting the overlap between the film shape in two consecutive snapshots, allowing for the extraction of additional information in each new frame. The process of determining this overlap consists of minimizing the differences in film thickness between two consecutive snapshots by shifting the pixels of the new frame pixel by pixel (varying *k*) until the minimum of Equation (27) is achieved. This operation can be expressed mathematically as:

(27) $$\min_{k}\left(\sum_{x=1}^{x=end}\left|h^{t+\Delta t}\left(x+k\right)-h^{t}\left(x\right)\right|\right)\;for\;k=1,2,3,\;\ldots$$

where $h^{t}\left(x\right)$ represents the film thickness in each *x*-pixel at time t, and $h^{t+\Delta t}\left(x\right)$ the film thickness in each *x*-pixel at time t+Δt. The variable k corresponds to the shift applied to the new frame that increases until the minimum sum of differences is reached. When the minimum value for the sum of differences in film thickness is achieved, it indicates that the two frames are optimally overlapped. This point determines the specific shifted position i, and the newly acquired information is accumulated in the final composite vector.

**Figure 19:**
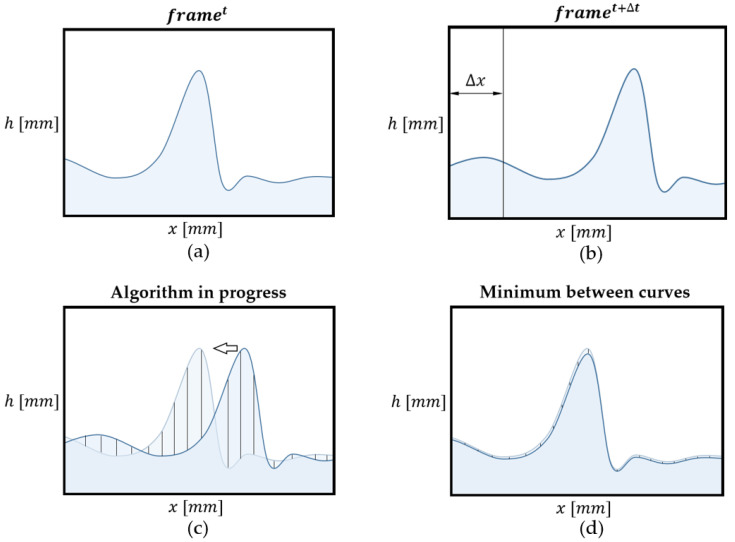
Visual representation of the algorithm to determine the new data of each consecutive frame. (**a**) frame at time *t*, (**b**) frame at time *t* + Δt, (**c**) algoritm to find k in progress, (**d**) comparison of both curves at the minimum.

The explanation is simple; if you have a film height $\;h^{t}\left(x\right)$ in the snapshot at time t at position *x* (expressed in number of pixels), you need to know where this same film height will be in the next snapshot at time t+Δt; this new position is denoted by x+k but k is not known. In other words, because the wave of Figure 19c is displaced several pixels, you need to know how many pixels k it is displaced this wave at time t+Δt from the same wave at time t. The algorithm designed finds *k* by minimizing Equation (27).

Figure 19 serves as a visual representation of the algorithmic process employed for the calculation described previously. It provides a step-by-step illustration of how the algorithm determines the spatial evolution of film thickness between two consecutive time steps, denoted as t and t+Δt. In Figure 19a, it is possible to observe the film thickness distribution at timestep t, while Figure 19b showcases the film thickness at the subsequent timestep, t+Δt. The objective of the algorithm is to precisely quantify the changes between these two frames by systematically analyzing corresponding points. The algorithm initiates this calculation by computing the total difference between equivalent points in Figure 19a,b. It does so by systematically shifting the film thickness curve of the frame at timestep t+Δt, as illustrated in Figure 19c. At each shift, the algorithm calculates the total difference between film thickness points, and this process is iteratively repeated for various pixel shifts. The algorithm continues these calculations, progressively shifting the frame at timestep t+Δt, until it reaches a point where the minimum total difference is achieved. At this juncture, the algorithm halts its operation, records the number of pixels it has moved (representing the optimal alignment) and stores the additional information acquired during this spatial evolution analysis into the composite final film thickness. This process is repeated for the subsequent pair of snapshots t+Δt and t+2Δt until all the images for each run are processed.

### 2.7. Uncertainty Analysis of the Measurements Performed by Conductance Probe and PLIF

The different equipment employed that contribute to experimental conditions uncertainties include the water flow meter (±0.3% of the maximum scale of 20 L/min) and the T-type thermocouples copper–constantan (biggest one of the following two values 1.0 °C or 0.75% of the measured temperature). The total error of the measurements usually entails an analysis of systematic error and accidental error. For the conductance probes, different devices contribute to the deviation due to systematic errors including the data acquisition system, the fitting error between the calibration curve and calibration points, and the positioning error during the calibration process. An extensive analysis in this regard has been carried out in previous experimental studies carried out by the group [8,9]. Accidental error requires the repetition of the experiments to account for deviations, usually following a normal distribution function. This error can be calculated by the Standard Error of the Mean using the next equation for a confidence level of 95% $\varepsilon_{acc}=t_{n-1}^{\alpha /2}\frac{s_{n}}{\sqrt{n}}$. As a reference, average errors estimated in previous studies for the conductance probe are 0.02 mm for the mean film thickness, 0.06 mm for the disturbance wave height and 0.42 Hz for the disturbance wave frequency.

The precise error assessment for PLIF techniques remains a topic with many open questions for the research community. The main errors are described in [31] and comprise two main errors: non-coherent interface error and total reflection error. The first error tends to underestimate the real interface while the second one leads to overestimation as explained in the Introduction and in Figure 5 of the current paper. These errors are consequently more relevant during conditions where the liquid film is subjected to high shear stresses (with high gas velocities). This study is limited to free fall conditions, reducing therefore the interface agitation and its influence. In addition, the high-speed camera is positioned at an angle of 40°, a placement where these errors are minimized according to [10]. The appearance of total internal reflection still can occur, particularly under steep changes in the film thickness. This error is very hard to estimate, and no current techniques can be employed to measure it. Nevertheless, study [7] shows that a median filter, as the one employed in this paper, with a proper window can suppress the influence of this error in the measurements. PLIF technique still has some open questions for error estimation, the reason why complementary methodologies are also being developed as S-PLIF or BBLIF, and further studies are needed. Similarly, as for the conductance probe, average errors estimated in previous studies by other authors [10] are about 5%, so maximum differences of around ±0.16 mm should be expected for all temperatures and Reynolds numbers.

## 3. Results and Discussion of the Experiments with the Conductance and the LIFT40 Methods at Different Liquid Reynolds Numbers and at Different Temperatures

This section of this study presents a comprehensive analysis of the experimental data obtained from the two distinct measurement techniques, PLIF and conductance probes, used to investigate annular two-phase flow. These techniques have been widely employed to study annular flow phenomena; however, a systematic comparison between them has been lacking in the literature. In this section, we provide a detailed examination of key parameters characterizing the liquid film, disturbance waves, and their temporal evolution, shedding light on the differences and similarities between the two measurement methods. Additionally, we discuss the implications of these findings and their significance in the context of annular flow characterization and engineering applications.

Several kinds of results have been obtained in the GEPELON facility when measuring the film thickness in the free-fall configuration for several Reynolds numbers of the downward water film and at three different temperatures 20 °C, 30 °C, and 40 °C. The measurements were performed using the conductance probe explained previously, with the three electrodes oriented perpendicularly to the flow direction as shown in Figure 9. Also, the PLIF40 measurements were performed for the same Reynolds numbers and temperatures, with the laser sheet and the CMOS camera located just above the conductance probe number 5 near the bottom of the test section where we have developed flow conditions. It is important to remark that the measurements performed using the PLIF technique and the ones carried out using the conductance probe were in regions very close but not the same. The distance between both measurement regions of 100 mm, and the region of the PLIF measurements is previous in time to the one of conductance measurements.

### 3.1. Comparative Results of the Experiments Performed with PLIF40 Concerning the Spatial and Temporal Resolution

Before comparing the PLIF and conductance probe, we analyzed the differences in the temporal evolution of the film thickness using the PLIF methodology. To determine the waveform, a minimum of one pixel resolution can be considered to generate the total evolution of the film thickness during the measured time. Nevertheless, to reduce the noise introduced by the binarization during the pre-processing of the images, a moving average filter with a window of 15 pixels has been applied. Therefore, the spatial resolution is slightly increased from 1 pixel (or 0.033 mm approximately) to 15 pixels, equivalent to 0.5 mm. The conversion relation is 1 mm equivalent to 30 pixels as explained in the previous section.

**Figure 20:**
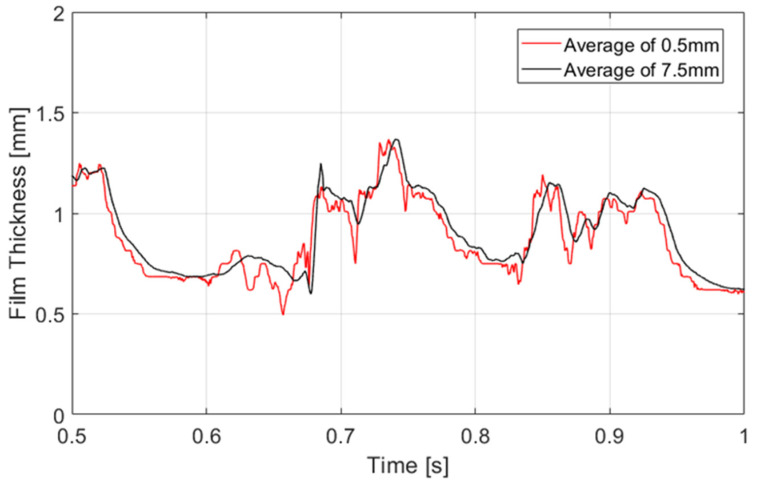
Comparison between the evolution of the film thickness with an average of 0.5 mm and an average of 7.5 mm.

Figure 20 shows a comparison of the waveform during 0.5 s between the processing of the film thickness considering 0.5 mm or 7.5 mm of spatial resolution. The reasoning behind taking 7.5 mm lies in the fact that the separation of the electrodes in the conductance probe is 6 mm with an electrode radius of 0.75 mm. Therefore, the total distance between the extremes of the emitter and received electrodes is 7.5 mm. It is possible to see in the figure that the average of 7.5 mm correctly reproduces the shape with a higher resolution but introduces subtle errors in the rugosity of the interface. As seen between 0.6 and 0.7 s in the graph, some ripple waves are neglected, reducing the capability to study them by methodologies with higher averaging.

**Figure 21:**
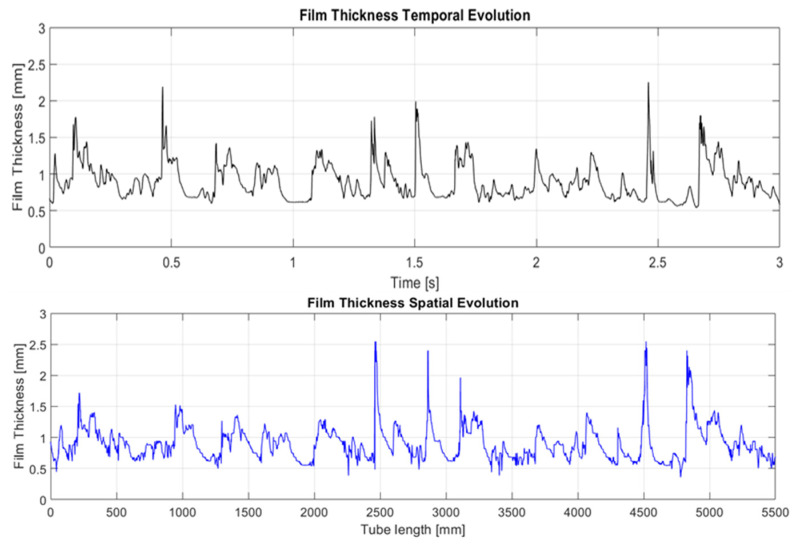
Comparison between temporal and spatial evolution of the film thickness.

Next, the results obtained from the analysis of the spatial and temporal evolution of the liquid film thickness in annular two-phase flow are presented. As discussed earlier, the spatial evolution methodology for the data gathered using PLIF differs from the temporal evolution. Figure 21 provides a comparison between these two distinct techniques employed to analyze the waveform of the liquid film. The first approach involves reconstructing the film thickness by iteratively adding new information obtained between frames, while the second method requires tracking a single pixel in each snapshot over time. Notably, this analysis brings to light the dynamic nature of the liquid film, where a 3 s interval translates to an equivalent spatial length of approximately 6 m due to the fast motion of the film. However, it is important to clarify that the scale of the *x*-axis in the spatial evolution representation is not a direct reflection of the physical length of the tube. Instead, it serves as a composite extension, if both the film thickness and the waves remain unchanged over time. This abstraction allows for a focused examination of the evolving patterns and behavior of the liquid film. Upon closer examination of the comparison between the two techniques, it becomes evident that both consistently identify the waveform. They exhibit similar trends, although subtle discrepancies manifest in most of the waves. Notably, the temporal evolution analysis showcases a more uniform pattern, while the spatial evolution analysis excels in detecting the rugosity present at the liquid–gas interface. Differences in the disturbance wave height are also detected. The first explanation for these differences is that the snapshots were performed at a rate of 1940.1 Hz, so the difference in time between snapshots was $0.51\times 10^{-3}\;s$. During this time, if the DW has a celerity, for instance, of 3 m/s, then travels 1.53 mm, which is equivalent to 45 pixels, so when capturing the temporal evolution at a given point, we are capturing points separated by approximately 45 pixels in space. This produces some differences when comparing spatial and time evolution as displayed in Figure 21.

**Figure 22:**
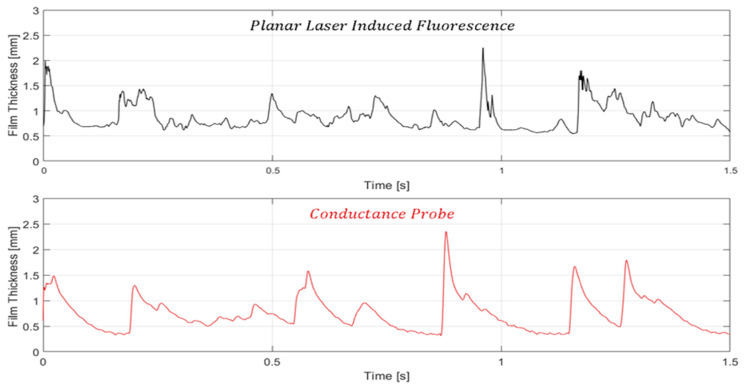
Comparison between temporal evolution of the film thickness for 1.5 s employing both techniques PLIF and conductance probe.

To compare the measurements between both techniques, the temporal evolution has been taken as a reference. It is important to underscore that the measurements conducted through the PLIF (planar laser-induced fluorescence) technique, and the conductivity probe were executed in not identical close regions, with a separation distance of approximately 100 mm. The measurement region using PLIF is, therefore, slightly anterior to the ROI of the probe. Figure 22 illustrates the temporal evolution of the liquid film through both techniques over a measurement period of 1.5 s. It is important to note that the spatial resolution plays an important role in the shape of the wave, as discussed before. The PLIF graph has been obtained with a spatial resolution of 0.5 mm while the conductance probe has a resolution of approximately 7.5 mm. Observable differences between the two techniques become apparent at first glance. Primarily, variations are discernible in the ripple waves. The spatial resolution of the camera allows for a much more detailed interface where a more detailed rugosity of the film is observed. Regarding the disturbance waves, subtle differences are also observed. For instance, in the latter two waves, a more pronounced dissociation is evident, meaning that even within the developed region, instantaneous interfacial characteristics continue to undergo temporal changes. It is worth emphasizing that transient statistics over time remain consistent, as explained in more detail in the following part of this discussion.

### 3.2. Comparative Results of the Experiments Performed with the PLIF40 at Different Reynolds Numbers of the Liquid, Different Temperatures and Different Spatial Resolutions

The experimental measurements using both methods were performed with the same boundary conditions, i.e., three different superficial velocities for the liquid $0.05\;\frac{m}{s},\;0.09\;\frac{m}{s}\;\mathrm{and}\;0.14\;\frac{m}{s}$, and three different temperatures of 20 °C, 30 °C and 40 °C for each superficial velocity. The Reynolds number of the liquid was calculated with the expression:

(28) $$Re_{L}=\frac{\rho_{l}J_{l}D}{\mu_{l}}=\frac{4\;\rho_{l}\;Q_{l}}{\pi \;D\;\mu_{l}}$$

with $J_{l}$ being the liquid superficial velocity, $\mu_{l}$ the dynamic viscosity, *D* the pipe diameter equal to 30 mm, and $Q_{l}$ the liquid flow rate.

**Figure 23:**
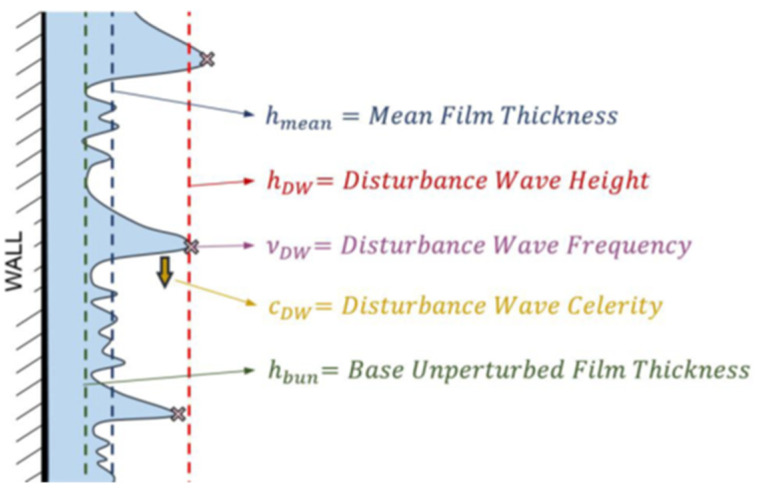
Most important variables of the liquid film.

Several magnitudes were calculated from the measurements in this paper and then compared for both types of measurement techniques, the average thickness of the liquid film $h_{mean}$, the average height of the disturbance waves $h_{DW}$, the average height of the ripple waves $h_{RW}$ and the average frequency of the disturbance waves $\nu_{DW}$. Figure 23 displays the most important average magnitudes of the annular flow.

To facilitate a fair evaluation, first, the PLIF results obtained have been compared between temporal measurements using the 7.5 mm average and the 0.5 mm average. It is important to remember that the measurements are the same, but the methodology used to capture the interface varies depending on the specific temporal average considered.

**Figure 24:**
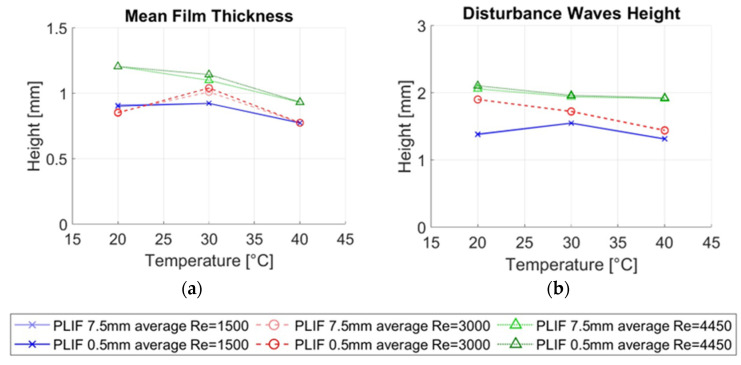
Comparison between 7.5 and 0.5 mm average for different temperatures and Reynolds numbers and their effect on the (**a**) mean film thickness, (**b**) disturbance waves height.

Figure 24a illustrates the mean film thickness $\left(h_{mean}\right)$ for the three temperatures under study and the different liquid Reynolds numbers. Each symbol marker and main color represents the different Reynold conditions. For each color, the lighter one is given to the 7.5 mm average while the darker color represents the 0.5 mm average. Among all the variables examined, minimal differences are observed in the mean film thickness between the two averaging methods, with data points being nearly identical. However, slight deviations are noted at 30 °C, likely attributed to minor height fluctuations in some waves, a phenomenon further explored in this section.

Figure 24b shows the height of the disturbance waves obtained for each case. Like the mean film thickness, both methods exhibit negligible deviations in wave height. Various authors have previously discussed the impact of obtaining averaged measurements across specific spatial resolutions [8]. Disturbance waves are large, coherent waves characterized by a flat plateau around their maximum height. As observed during the PLIF processing, these waves often extend over a considerable distance, occasionally spanning the entire PLIF measurement region (35 mm). It appears that an average of 7.5 mm does not significantly affect the detection of the height of these types of waves. However, during the propagation of disturbance waves, small ripple waves may be superimposed onto the flat plateau. While the average height remains unchanged, subtle roughness may appear at the interface, which can only be discerned with an appropriate spatial resolution. Ripple waves are, therefore, more intricate to study, given the challenges involved in their detection and analysis.

**Figure 25:**
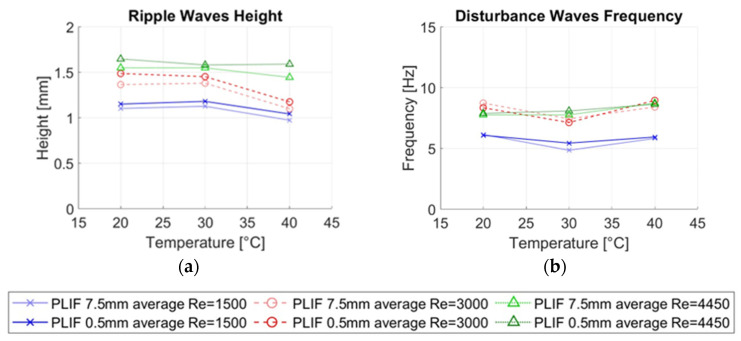
Comparison between 7.5 and 0.5 mm averaging for different temperatures and their effect on the (**a**) ripple wave height, (**b**) disturbance wave frequency.

Figure 25a illustrates the ripple wave height obtained for both spatial resolutions using PLIF40. In this study, we have not considered the valleys of the ripple waves; hence, the values represent the height from the wall. For this variable, more significant differences are observed, with a deviation of approximately 10% between the two averages, primarily due to their smaller size.

In Figure 25b, the frequency of disturbance waves has been represented. Very slight deviations are observed, likely stemming from the wave detection algorithm. The introduction of greater roughness may lead to the inclusion of waves that might not have been considered previously, exerting a subtle influence on the frequency counting. However, these deviations all fall within a range of a maximum of 5%, with most of them registering below 3%.

### 3.3. Comparative Results of the Experiments Performed with the Conductance Probe and PLIF40 at Different Reynolds Numbers of the Liquid and Different Temperatures

The final goal of this study is to analyze the differences observed in all the variables under investigation when comparing PLIF40 and the conductance probe. Building on the preceding discussion concerning the spatial resolution of PLIF40, we have employed a 7.5 mm spatial processing approach for the purpose of comparison. This approach ensures that we consider the spatial average affecting the conductance probes, specifically spanning the 7.5 mm distance between the extremities of the emitter and receiver electrodes.

**Figure 26:**
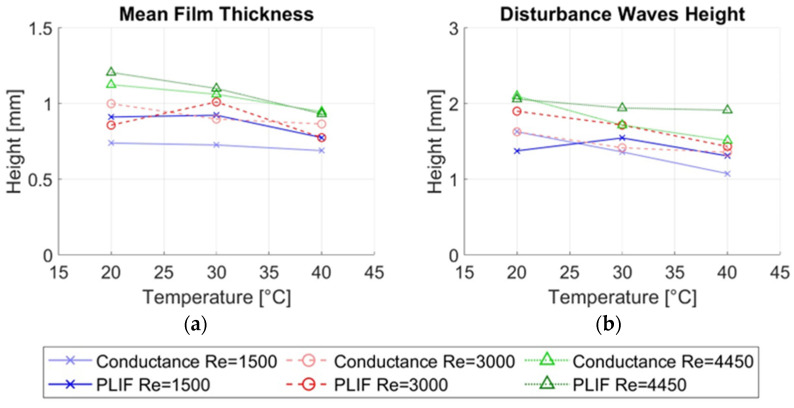
Comparison between conductance probe and PLIF40 techniques for different temperatures and their effect on the (**a**) mean film thickness, (**b**) disturbance wave height.

Figure 26a shows the mean film thickness measured with both techniques as the main colors and markers of different liquid Reynolds numbers. Within each color, the light one corresponds to the conductance probe and the darker one to the PLIF40. It is observed that the mean film thickness measured with both techniques decreases when the temperature increases. In addition, it is also observed that the differences in the average thickness measured with both techniques diminish when the temperature increases. So, at the highest Reynolds number 4500 and the highest temperature 40 °C both techniques give the same result for the average thickness. Because the water conductivity increases with the temperature changes in conductance are more easily detected at higher temperatures, the conductance technique is more sensitive to temperature than LIF. In addition, it is observed with both techniques that the mean film thickness increases with the Reynolds number at all temperatures.

The disturbance wave height comparison is depicted in Figure 26b. In general, the height of the DW decreases with the temperature for both measuring techniques as observed in Figure 26b; however, the changes in the height of the DW with temperature are more pronounced in the conductance probe than in the LIF method due to the high dependence of the conductivity with the temperature. Also, it is observed that the height of the DW increases with the Reynolds number at all the temperatures with both techniques.

**Figure 27:**
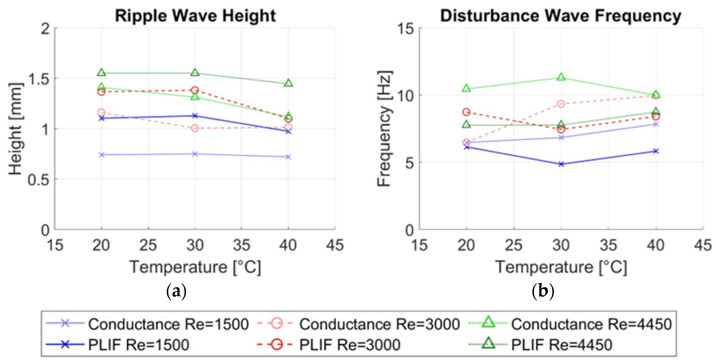
Comparison between conductance probe and PLIF40 techniques for different temperatures and their effect on the (**a**) ripple wave height, (**b**) disturbance wave frequency.

Finally, we need to discuss the differences found in the ripple wave height. As shown in Figure 27a, the average height of the ripple waves is smaller when measured with the conductance probe at all temperatures and Reynolds numbers than the one measured with the LIF40 method, even though we use an average of 7.5 mm for the LIF40 method. The distance of 7.5 mm was chosen equal to the maximum distance between the extremes of the electrodes, but because the electric field can have some curvature before leaving and penetrating the emitter and receiver electrodes, the averaging performed in the conductance probe could span a bigger distance in the transversal and flow directions than that spanned by the electrodes diminishing the average height for small waves. An additional reason why the measured height of the RW by the conductance probe is smaller is that the ripple waves are not coherent in the circumferential direction, and because the size of this wave is smaller than the one of the DW, then the region occupied by the sensing region of the conductance probe (where the wave is averaged) is bigger than the region occupied by the RW; as a consequence, the height measured by the conductance probe is smaller than the real height.

## 4. Conclusions

In this paper, the GEPELON facility was employed to conduct a study of air-water annular two-phase flow. The primary objective was to investigate the behavior of this flow using different techniques under varying conditions, specifically focusing on film thickness measurement in a free-fall configuration. This study included a range of Reynolds numbers for the downward water film and was conducted at three distinct temperatures: 20 °C, 30 °C and 40 °C. The two measurement techniques employed are the conductance probe, featuring three electrodes oriented perpendicularly to the flow direction, and the PLIF40 method, employing a laser sheet and a high-speed camera. The test measurements were performed in regions close to each other but not identical, with a separation of 100 mm being the PLIF measurements before, as displayed in Figure 8.

For conductance probe measurements, we employ a method based on the current between emitter and receiver electrodes at high frequencies located flush mounted to the wall. This approach allows for precise measurement of temporal variations in film thickness with a high sampling rate and high recording times. The technique relies on the difference in conductivity between liquid and gas, employing three electrodes embedded in the wall to measure the liquid film thickness. In PLIF optical measurements, a fluorescent dye is added to the water, making the liquid film emit light under a laser sheet. However, challenges arise due to errors caused by refraction and total reflection at the gas–liquid interface. To address this, we employ optical corrections involving taking the measurements with an angle of 40°, the use of optical filters and a correction algorithm based on known refractive indices of the media. Sub-pixel edge detection is utilized to precisely locate the gas–liquid interface in PLIF measurements. The Sobel operator is employed for edge detection, enhancing the accuracy of the measurements.

To analyze the temporal evolution of film thickness, data processing techniques are applied to both conductance probe and PLIF measurements. For PLIF, this involves calculating the temporal evolution of the film thickness within different windows and identifying waves in the signal. A standardized approach is adopted to make comparisons between the two measurement techniques meaningful. Furthermore, spatial evolution analysis of film thickness in PLIF measurements is conducted by an algorithm able to systematically compare consecutive snapshots and detect changes between them. This process enables a detailed understanding of how the film thickness evolves over time with a very high spatial resolution.

The comparative analysis of results begins with an examination of the differences in the temporal evolution of film thickness using the PLIF methodology. The spatial resolution of PLIF was found to impact the accuracy of waveform reproduction. A 0.5 mm spatial resolution offered higher precision but introduced subtle errors in interface rugosity. Conversely, a 7.5 mm spatial resolution provided a more uniform representation but dampened the visualization of smaller ripple waves.

Subsequently, a study of the experiments conducted with PLIF40 at different Reynolds numbers and temperatures is shown in Section 3. Several key parameters, including average film thickness ($h_{mean}$), disturbance wave height ($h_{DW}$), ripple wave height ($h_{RW}$) and disturbance wave frequency ($\nu_{DW}$), were calculated. Comparative analysis of results obtained with different spatial resolutions within PLIF (0.5 mm and 7.5 mm) revealed minimal differences in mean film thickness and disturbance wave height. However, when investigating ripple waves, characterized by smaller size and interface roughness, a 10% deviation between spatial resolutions was observed. Disturbance wave frequency exhibited only slight deviations, primarily within a 3–5% range.

The final goal of the study was to compare results obtained using the conductance probe and PLIF40 techniques. A 7.5 mm spatial processing approach was applied to PLIF40 to match the spatial averaging effect of the conductance probe. The comparison included the same main key film characteristics. For mean film thickness, both techniques produced consistent trends across various liquid Reynolds numbers and temperatures, with minor differences at 30 °C. As the Reynolds number of the liquid film increases, a growth in the mean film thickness is observed at all temperatures. However, when the temperature rises, the average film thickness diminishes for all Reynolds numbers. Additionally, the differences in average thickness measured by both methods diminished when the temperature rose, ultimately converging at the highest Reynolds number and temperature. This convergence highlights the conductance technique’s heightened sensitivity to temperature variations due to changes in water conductivity.

Furthermore, disturbance waves, being large and coherent, were not significantly affected by spatial averaging, and both methods yielded similar wave height results. Both techniques also showcased a decrease in disturbance wave height with rising temperatures. However, this reduction was more pronounced when using the conductance probe, primarily due to the strong dependence of conductivity on temperature. In contrast, ripple waves, characterized by their smaller size and interface roughness, showed more substantial differences, with a maximum of 28% deviation between techniques. Disturbance wave frequency also showed deviations, primarily due to the wave detection algorithm and the short PLIF40 total time of each run, limiting its capacity for transient statistics.

This research provides insights into annular two-phase flow behavior measurement techniques. Both PLIF40 and the conductance probe prove valuable for studying film characteristics, each with its unique strengths and limitations. The choice between these two techniques relies on the specific aspects of the liquid film under investigation. PLIF40, with a 0.5 mm spatial resolution or less, excels in capturing interface rugosity and studying smaller ripple waves. In contrast, the conductance probe offers consistent and reliable results, especially for averaged parameters such as mean film thickness and disturbance wave height and frequency. These findings contribute to a deeper understanding of annular two-phase flow dynamics and offer researchers valuable options for measurement techniques, adapted to their research objectives and desired level of detail.

## Data Availability

Data are available upon request.

## Nomenclature

**Acronyms**	
DW	Disturbance Waves
RW	Ripple Waves
PCB	Printed Circuit Board
LIF	Laser-induced Fluorescence
BBLIF	Brightness-based Laser-induced Fluorescence
PLIF	Planar Laser-induced Fluorescence
PLIF40	Planar Laser-induced Fluorescence at 40° angle
PSD	Power Spectral Density
ROI	Region of Interest
FEP	Fluorinated Propylene–Ethylene
PFA	Perfluoroalkoxy alkanes
GEPELON	Facility Acronym of *Generación de Película Ondulatoria*
SMR	Small Modular Reactor
DAQ	Data Acquisition
ID	Internal Diameter
TIFF	Tagged Image File Format
**Variables**	
$\lambda_{exc}$	Excitation wavelength
$\lambda_{emiss}$	Emission wavelength
Re	Reynolds number
$f_{PSD\;}$	Power Spectral Density Frequency
α	Absorption coefficient of fluorescent light
K	Interfacial reflection index between phases
C(x, y)	Compensation matrix for BBLIF technique
h	Film thickness
$h_{meas}$	Film thickness measured by the high-speed camera
$h_{r}$	Real film thickness
$h_{mean}$	Mean film thickness
$h_{DW}$	Disturbance wave height from the wall
$h_{RW}$	Ripple wave height from the wall
$\nu_{DW}$	Disturbance wave frequency
$h^{t}\left(x\right)$	Film thickness at t time depending on each *x* in a snapshot
$y_{S}$	Viscous permeability of porous material
$V_{sat}{}^{scaled}$	Saturation voltage at operation conditions
$V_{sat}{}^{b}$	Saturation voltage at base calibration conditions for 20 °C
$V_{i}{}^{scaled}$	Voltage for each point of the calibration curve at operation conditions
$V_{i}{}^{b}$	Voltage for each point of the calibration curve at base conditions for 20 °C
$V_{min}{}^{scaled}$	Minimum voltage of the calibration curve at operation conditions
$V_{min}{}^{b}$	Minimum voltage of the calibration curve at base conditions for 20 °C
$n_{w}$	Refractive index of water
$n_{p}$	Refractive index of the tube
$n_{a}$	Refractive index of the air
$G_{x}$	Sobel horizontal operator
$G_{y}$	Sobel vertical operator
G	Gradient of the Sobel operator
k	Increasing index to account for the pixel shift in the algorithm for film thickness reconstruction
$j_{l}$	Superficial velocity of the liquid
